# 3D Microtumors Representing Ovarian Cancer Minimal Residual Disease Respond to the Fatty Acid Oxidation Inhibitor Perhexiline

**DOI:** 10.1002/adhm.202404072

**Published:** 2025-02-09

**Authors:** Xingyun Yang, Mara Artibani, Yongcheng Jin, Aneesh Aggarwal, Yujia Zhang, Sandra Muñoz‐Galvan, Ellina Mikhailova, Lena Rai, Nobina Mukherjee, Ravinash Krishna Kumar, Ashwag Albukhari, Shaohua Ma, Linna Zhou, Ahmed Ashour Ahmed, Hagan Bayley

**Affiliations:** ^1^ Department of Chemistry University of Oxford Oxford OX1 3TA UK; ^2^ Ovarian Cancer Cell Laboratory MRC Weatherall Institute of Molecular Medicine University of Oxford Oxford OX3 9DS UK; ^3^ Nuffield Department of Women's & Reproductive Health University of Oxford Oxford OX3 9DU UK; ^4^ Institute of Electrical and Microengineering École Polytechnique Fédérale de Lausanne Lausanne 1015 Switzerland; ^5^ Instituto de Biomedicina de Sevilla IBiS/Hospital Universitario Virgen del Rocío/CSIC/Universidad de Sevilla Avda Manuel Siurot Seville 41013 Spain; ^6^ Department of Infectious Disease Imperial College London South Kensington London SW7 2AZ UK; ^7^ Biochemistry Department Faculty of Science King Abdulaziz University Jeddah 21589 Saudi Arabia; ^8^ Tsinghua Shenzhen International Graduate School (SIGS) Tsinghua University Shenzhen 518055 China; ^9^ Tsinghua‐Berkeley Shenzhen Institute (TBSI) Tsinghua University Shenzhen 518055 China; ^10^ Ludwig Institute for Cancer Research Nuffield Department of Medicine University of Oxford Oxford OX3 7DQ UK

**Keywords:** 3D cancer models, drug testing platforms, microfluidics, minimal residual disease, ovarian cancer

## Abstract

The poor survival of ovarian cancer patients is linked to their high likelihood of relapse. In spite of full apparent macroscopic clearance, tumor recurrences arise from cells that are resistant to primary chemotherapy in the form of minimal residual disease (MRD). MRD exhibits distinct molecular drivers from bulk cancer and therefore necessitates alternative therapeutic strategies. However, there is a lack of 3D models that faithfully recapitulate MRD *ex vivo* for therapy development. This study constructs microfluidics‐based 3D microtumors to generate a clinically‐relevant model for ovarian cancer MRD. The microtumors recapitulate the non‐genetic heterogeneity of ovarian cancer, capturing the “Oxford Classic” five molecular signatures. Gene expression in the 3D microtumors aligns closely with MRD from ovarian cancer patients and features the upregulation of fatty acid metabolism genes. Finally, the MRD 3D microtumors respond to the approved fatty acid oxidation inhibitor, perhexiline, demonstrating their utility in drug discovery. This system might be used as a drug‐testing platform for the discovery of novel MRD‐specific therapies in ovarian cancer.

## Graphical abstract

Minimal residual disease (MRD) leads to cancer relapse and poor survival in ovarian cancer. The lack of representative 3D models limits therapy development. The microfluidics‐based high throughput 3D microtumor platform described here generates clinically‐relevant MRD models. The 3D microtumors recapitulate the molecular signatures of MRD from patients. This platform can be used to advance the understanding of MRD and test novel treatments.

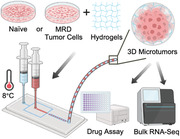

## 1 Introduction

Drug resistance is responsible for up to 90% of cancer‐related deaths.^[^ ^1^ ^]^ A particularly challenging group of treatment‐resistant cells is represented by Minimal Residual Disease (MRD), the microscopic clusters of malignant cells that remain in patients after complete clinical/radiological response and are capable of reinitiating tumors.^[^ ^2^ ^]^ Investing in therapeutics that specifically target MRD could help delay or altogether prevent relapses, moving us a step closer to the chronic management or potential eradication of cancer.^[^ ^3^ ^]^

However, we lack deep knowledge of MRD biology as well as appropriate experimental models that could be used as platforms for testing effective compounds, especially in solid tumors.

One of the very few exceptions is ovarian cancer,^[^ ^4^ ^]^ for which we recently obtained the first transcriptomic characterization of clinical MRD. These cancer lesions were biopsied from “exceptional responders”, patients who responded so well to primary chemotherapy that only microscopic tumor foci were found during the interval debulking surgery. We compared these MRD cells to the chemotherapy‐resistant cells obtained from “poor responders”, women that showed extensive macroscopic disease after primary treatment. Although both cell populations survived chemotherapy, we observed significant transcriptional differences between them, highlighting the need for distinct therapeutic approaches to target each population.

Our current objective is to develop a clinically relevant model of MRD for drug testing, enabling us to specifically evaluate drugs that target MRD‐specific pathways. We considered mouse, 2D, and 3D models, all of which have different balances of feasibility, accuracy, and cost.

Although mouse models exist to study metastatic ovarian cancer, their reproductive physiology^[^ ^5^ ^]^ as well as omental anatomy (one of the most common MRD sites)^[^ ^6^ ^]^ differ from humans and this species does not develop spontaneous ovarian tumors. Conventional 2D cell culture has been a standard in vitro model for decades. However, under these conditions, cell morphologies and bioactivities deviate from those found in vivo,^[^ ^7^ ^]^ which can deeply affect response to chemotherapeutics. For instance, a transcriptomic study of single cell‐derived spheroids from ovarian cancer ascites has shown that although 2D monolayers support proliferation and tumor growth cascades, 3D spheroids additionally capture aspects of cholesterol and lipid metabolism, which are features of metastatic disease.^[^ ^8^ ^]^ These pathways are implicated in the lipid signature we observed in ovarian cancer MRD.^[^ ^4^ ^]^ Therefore, in this context, 3D models are essential for drug discovery.

Moreover, cancers typically organize in 3D, creating a hypoxic setting with intimate intercellular signaling, and ultimately achieve anchorage‐independent growth. 3D cancer cultures offer extensive cell‐cell and cell‐ECM (extracellular matrix) interactions,^[^ ^9^ ^]^ and preserve the cell polarity, morphology, gene expression, and topology seen in vivo.^[^ ^10^ ^]^ They also offer the prospect to co‐culture vascular and stromal elements to investigate heterotypic interactions.^[^ ^11^ ^]^ This is a crucial prerequisite for our model, since environment‐mediated drug resistance is a major contributor to MRD.^[^^]^

3D cell culture technologies can be classified as scaffold‐free or scaffold‐based. The former include multi‐cellular aggregates formed using the hanging‐drop method,^[^ ^13^ ^]^ suspension plates,^[^ ^14^ ^]^ silicone micro‐molds,^[^ ^15^ ^]^ or spinner flasks.^[^ ^16^ ^]^ The latter employ biocompatible materials, such as hydrogels, as structural supports for cell culture.^[^ ^10^, ^11^ ^]^ Cells proliferate in the scaffolds and establish cell‐cell and cell‐ECM interactions, displaying natural 3D structures instead of flattening out as they do in 2D culture.^[^ ^17^ ^]^ Despite all these advantages, the application of 3D cell culture models in drug testing has been constrained by long fabrication times, great size variability, poor repeatability, and low productivity.^[^ ^10a^ ^]^

Microfluidics is a promising tool for dealing with various unmet needs in 3D cell culture. Based on the immiscibility of aqueous and oil phases, discrete aqueous droplets of uniform size and composition can be generated by microfluidics, in which cells can be encapsulated in a highly reproducible and high‐throughput manner for subsequent 3D culture. Microfluidics has already been adopted to create organoids or tumor spheroids to predict drug responses,^[^ ^18^ ^]^ investigate tumor vascularization,^[^ ^19^ ^]^ and study hair follicle regeneration with stem cell patterning.^[^ ^20^ ^]^ Ding et al. reported the development of micro‐organospheres derived from colorectal cancer patients using a microfluidics platform and successfully predicted patient outcomes in response to drug treatments with high accuracy.^[^ ^21^ ^]^ These findings suggest that microfluidics‐based 3D cell models represent a highly effective tool for clinical research and personalized medicine.

In the present work, a microfluidics platform was established to fabricate 3D microtumors, with readily customizable size, morphology, and hydrogel choice. First‐line chemotherapeutics testing demonstrated the utility of this system in pharmacology. 3D microtumors showed the same molecular signatures observed in clinical MRD and were successfully used as a drug testing platform, which led not only to the identification of a very promising therapeutic agent but also to significant insight into resistance mechanisms.

## 2 Results

### 2.1 Microfluidics Platform Generates 3D Microtumors of Tailorable Size, Cell Content, and Shape

**Figure 1:**
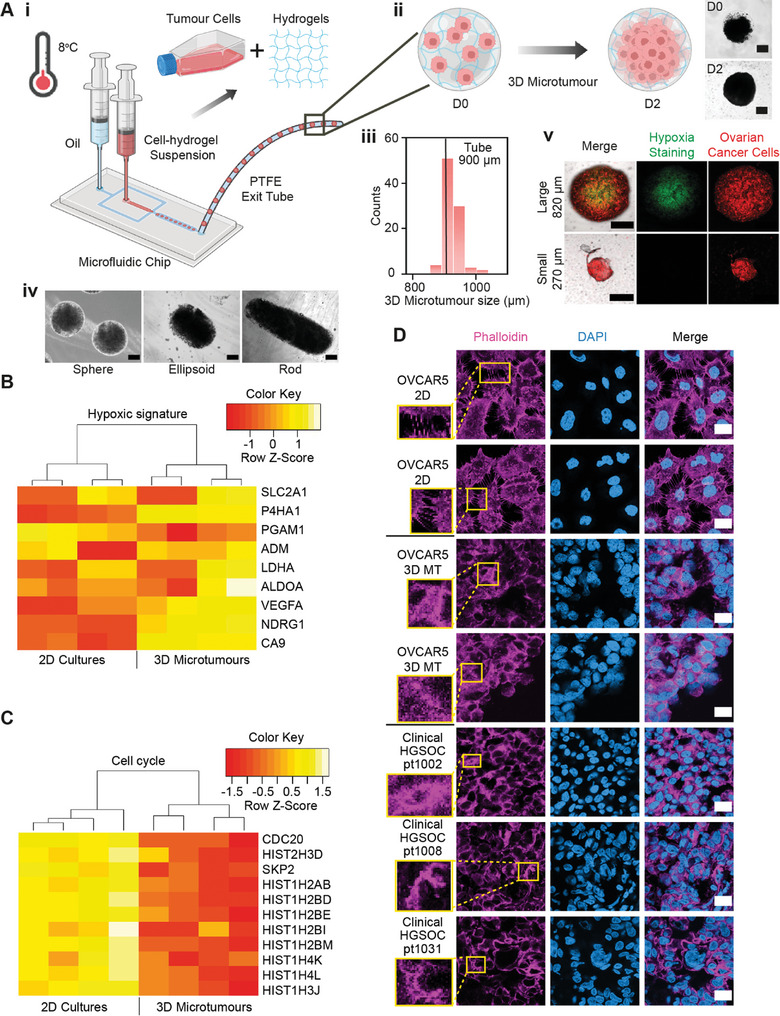
3D microtumors are tailored to recapitulate important features of in vivo cancer cells. (Ai, ii) 3D microtumors fabricated with the microfluidics platform. (Aiii) Size distributions of 3D microtumors composed of OVCAR‐5/RFP cells and Matrigel. (Aiv) Bright field microscope images of different shapes of 3D microtumors taken on D0 of fabrication. The shapes of sphere, ellipsoid, and rod were achieved by adjusting the cell‐hydrogel suspension:oil flowrate ratio to 1000:3000, 1000:1000, and 1000:300 µL h^−1^, respectively. The ellipsoid‐shaped microtumors were used for the rest of the experiments. Scale bar = 300 µm. (Av) Confocal images of hypoxia staining (green) for large and small 3D microtumors. (B,C) Heatmaps showing the expression of (B) hypoxic signature and (C) cell cycle‐related genes in 2D and 3D cultures of OVCAR5. (D) Confocal images of 2D cultures (OVCAR5), 3D microtumors (3D MT, OVCAR5) at D10, and clinical High‐Grade Serous Ovarian Cancer (HGSOC) samples stained with phalloidin (pink), DAPI (blue). Scale bar = 20 µm. Inset: zoom‐in images for regions of interest stained with phalloidin.

3D microtumors, tumor cells encapsulated in biocompatible hydrogels, were created using a surfactant‐free, droplet‐based microfluidics platform. Microfluidic fabrication requires only 3 min to produce 100 microtumors, and is followed by a gelation time dependent on the hydrogel used (**Figure** **1**Ai; Figure S1A, Supporting Information). To test the versatility of our platform, we used different hydrogels including Matrigel, collagen, agarose, and silk fibroin, which have been successfully used for different cell types. For example, agarose can be used to fabricate 3D bacterial colonies.^[^ ^22^ ^]^ Matrigel was ultimately chosen for the fabrication of MRD microtumors due to its ability in producing 3D cultures of cancer cells, including cancer organoids. We have successfully fabricated 3D microtumors from a wide range of both cancer and normal tissue cell lines using various hydrogels (Table S1 and S2 and Figure S1B, Supporting Information). Cells proliferated within the structures and increased their density by D2 (Figure 1Aii), and long‐term culture of 3D microtumors for up to 9 weeks has been achieved (Figure S1C, Supporting Information).

Importantly, the microfluidics fabrication process does not cause any damage to the cells, as shown by high cell viability of >90% (Figure S1D and Table S3, Supporting Information). Moreover, our platform offers flexibility in adjusting the size of the 3D microtumors (by simply using tubes with different inner diameters, Figure 1Aiii), their cell content (by adjusting the ratio of different cell types when preparing the cell‐hydrogel suspension), and shape (by varying the flowrate ratio of cell‐hydrogel suspension to oil, Figure 1Aiv). All the different sizes we fabricated showed a narrow size distribution with a 2–6% coefficient of variation (Figure S1E, Supporting Information and **Table** **1**), which is essential for reliable and reproducible drug testing experiments.

**Table 1:** Dimensions of 3D microtumors formed from Matrigel and OVCAR‐5/RFP tumor cells, 3T3 fibroblast cells, and a 50:50 mixture of OVCAR‐5/RFP cells and 3T3 cells (co‐culture). Size was measured on D0 of fabrication as described in the Experimental Section 'Characterizations of 3D Microtumors‐Size Distribution'.

Cell	Size group	3D microtumor size (µm)			
		N total	Median	Mean ± SD^a)^	CV^b)^
OVCAR‐5/RFP	Small	84	320	320 ± 7.2	2.3%
OVCAR‐5/RFP	Medium	77	670	670 ± 20	3.0%
OVCAR‐5/RFP	Large	90	920	930 ± 25	2.7%
Co‐culture	Small	88	300	300 ± 7.5	2.5%
Co‐culture	Medium	76	680	680 ± 40	5.9%
Co‐culture	Large	86	910	920 ± 26	2.8%
3T3	Small	62	310	320 ± 12	3.8%
3T3	Medium	74	700	700 ± 23	3.3%
3T3	Large	84	990	990 ± 28	2.8%

^a)^ SD: standard deviation;

^b)^ CV: coefficient of variation. CV = SD/Mean.

### 2.2 3D Microtumors Recapitulate Key Physiological Features of Tumors

Certain physical and biochemical characteristics of tumor cells (such as low oxygen tension, cytoskeleton organization, and a clinically relevant dose‐response to drugs) are particularly difficult to recreate in vitro, which leads to the use of sub‐optimal models for drug testing and eventually disappointing results from clinical trials.^[^ ^6^ ^]^

Hypoxia contributes to reshaping the tumor microenvironment and the development of immunosuppression and chemoresistance,^[^ ^23^ ^]^ with hypoxic cores commonly observed in tumors with diameters larger than 400–500 µm.^[^ ^24^ ^]^ While 2D monolayer cells lack the gradients of oxygen required to produce hypoxia, the initial size of microtumors prepared by prevailing methods usually falls within the range of only 100–300 µm.^[^ ^16^, ^24^, ^25^ ^]^ In the present work, we produced 3D microtumors with larger initial sizes (>800 µm), and observed hypoxic cores just 1 day after fabrication (Figure 1Av). 3D spheroids prepared with other methods, such as the forced aggregation method, required 11–21 days to grow to a comparable size and generate hypoxic cores (Table S4, Supporting Information).^[^ ^24^, ^25^ ^]^ We also confirmed the expression of key hypoxia genes (P4HA1, VEGFA, NDRG1) through immunofluorescence (Figure S2A,B, Supporting Information) in older microtumors (D10), where the hypoxic core had expanded.

RNA‐Seq on both 2D cultures and 3D microtumors confirmed that, regardless of the cell line used, the microtumors overexpressed genes associated with several biological processes related to hypoxia (such as a 16‐fold enrichment for the positive regulation of VEGF production) (Figure S2C, Supporting Information). We also analyzed the expression of an ovarian cancer‐specific hypoxic signature^[^ ^26^ ^]^ and saw that almost all the genes were upregulated in the OVCAR5 microtumors (Figure 1B).

On the other hand, 2D cultures were enriched for cell cycle‐related genes (Figure 1C; Figure S2D, Supporting Information), consistent with the faster cell division supported by the continuous supply of nutrients and oxygen in monolayer systems.

Another key feature of tumor cells is the organization of their cytoskeleton, which plays a crucial role in cell motility, and therefore, in invasion and metastasis. Specifically, actin filaments can act at different levels, from providing a connection with the ECM, to being mechanosensors and signaling scaffolds,^[^ ^27^ ^]^ all of which are altered in cancer.

Several studies have reported that the actin patterns and dynamics observed in living tumors are not recreated in 2D cultures or most 3D systems,^[^ ^28^ ^]^ especially when it comes to stress‐fiber structures.^[^ ^29^ ^]^ To investigate whether our microtumors recapitulated the actin distribution observed in clinical ovarian cancer, we used phalloidin staining on OVCAR5 cells grown in 2D cultures, OVCAR5 3D microtumors and biopsies of High‐Grade Serous Ovarian Cancer (HGSOC) before chemotherapy (Figure 1D). While the 2D cells are contained many thin filipodia, a rich network of thick stress‐fiber‐like actin bundles is present in both 3D microtumors and HGSOC samples (which were also stained for E‐cadherin to rule out stromal contamination, Figure S3, Supporting Information).

**Figure 2:**
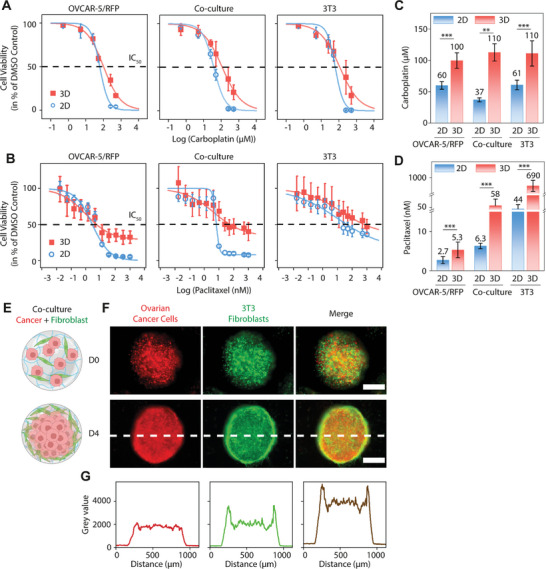
Dose‐response of 3D microtumors fabricated by the microfluidics platform. (A,B) Dose‐response curves of 3D microtumors and 2D cells treated with serial dilutions of carboplatin (A) and paclitaxel (B). For each drug concentration, 3D microtumors *n* = 15–20 (each microtumor was treated with drug individually to generate drug response data) and 2D cultures *n* = 12–14 from 3 to 4 independent experiments. (C,D) Bar graphs of IC_50_ values calculated from the dose‐response curves for carboplatin (C) and paclitaxel (D). Significance was tested using a Tukey test. ***p < 0.001, **p < 0.01. (E) Schematic diagram of co‐culture 3D microtumors composed of tumor cells and fibroblasts at D0 and D4. The diagrams were created with BioRender.com. (F) Epifluorescence images of co‐culture 3D microtumors (50:50 mixture of OVCAR‐5/RFP and 3T3 fibroblasts) on D0 and D4 after fabrication. Scale bar = 300 µm. (G) Fluorescence intensity profiles along the white dashed line across a co‐culture 3D microtumor on D4. Left for OVCAR‐5/RFP (red), middle for 3T3 fibroblasts (green), and right for merge (brown).

In terms of response to chemotherapeutic agents, we observed that 3D microtumors showed higher resistance compared to 2D cells for both carboplatin and paclitaxel (**Figure** **2**A–D; Figure S4, Supporting Information), with IC_50_ values of 100 µm (carboplatin) and 5.3 nm (paclitaxel) for 3D microtumors, and 60 µm (carboplatin) and 2.7 nm (paclitaxel) for 2D cells (Table S5, Supporting Information).

In the clinic, carboplatin and paclitaxel are administered intravenously every 3 weeks for eight cycles. The dosage of carboplatin is calculated to deliver an area under curve (AUC) of 5 (mg mL^−1^)⋅min, giving a theoretical maximum plasma concentration (C_max_) of 280 µm in a typical patient (Table S6, Supporting Information), although the true C_max_ is ≈115 µm.^[^ ^30^ ^]^ in vivo, ≈50% reduction in the CA125 biomarker is observed with each neoadjuvant chemotherapy cycle.^[^ ^31^ ^]^ This response aligns to the IC_50_ for carboplatin in 3D microtumors while 2D monolayers are around twice as sensitive. With this said, recapitulating pharmacokinetics is difficult; the in vitro carboplatin dose to deliver the same AUC as in vivo would be just 6.5 µm. Moreover, in vivo a long‐lived plasma paclitaxel concentration above 50 nm is what is typically associated with clinical efficacy – ten‐fold higher levels than required in vitro in 2D.^[^ ^32^ ^]^

Stromal cells, such as fibroblasts, are crucial components in the ovarian cancer microenvironment and can regulate tumor progression.^[^ ^33^ ^]^ Therefore, we incorporated fibroblasts in the ovarian cancer 3D microtumors (co‐culture, a 50:50 mixture of OVCAR‐5/RFP tumor cells and 3T3 fibroblasts) to provide a more physiological relevant microenvironment. When treated with paclitaxel, a 11‐fold higher IC_50_ value was found in co‐culture than with OVCAR‐5/RFP tumor cells alone 3D microtumors. While for 2D cultures, the IC_50_ was only 2.3‐fold higher in co‐culture than in tumor cells only (Figure 2C,D, Table S7, Supporting Information). These IC_50_ values indicate that crosstalk between fibroblasts and tumor cells is different in 3D and 2D cultures, which may contribute to therapeutic failure.

Intriguingly, distinct migration patterns were observed for co‐culture 3D microtumors. OVCAR‐5/RFP tumor cells and 3T3 fibroblast cells were well‐mixed before fabrication and both cell types were homogeneously dispersed throughout the 3D microtumors on D0. The 3T3 fibroblasts started to migrate toward the periphery on D2 and clearly accumulated at the edge of the structure by D4. Conversely, OVCAR‐5/RFP tumor cells remained evenly distributed within the 3D microtumor from D0 to D4 (Figure 2E–G). Similar core‐shell structures in co‐cultures of breast cancer cells with fibroblasts^[^ ^34^ ^]^ or epithelial cells^[^ ^35^ ^]^ have been previously reported. The core‐shell structure may also contribute to the increased IC_50_ values of co‐culture 3D microtumors compared to those composed of tumor cells only.

### 2.3 3D Microtumors as a Superior Model of Ovarian Cancer MRD

One of the biggest challenges in cancer research is finding new therapeutics that can eradicate chemotherapy‐resistant cells. This problem is especially relevant in ovarian cancer, which exhibits a high recurrence rate of >80% within 18 months^[^ ^36^ ^]^ due to MRD, drug‐resistant cells that survive first‐line treatment and initiate relapse (**Figure** **3**A).

Figure 33D microtumors as a superior model of ovarian cancer MRD. (A) Schematic diagram of the standard clinical management of patients with ovarian cancer. (B) Principal component analysis plots of RNA‐Seq data showing information from 2D cultures, 3D microtumors, and the previously published libraries obtained from patients with MRD. (C) Number of differentially expressed genes (DEGs) between MRD 2D cultures or MRD 3D microtumors compared to clinical samples. The diagrams were created with BioRender.com. (D,E) Overlap of DEGs in MRD 2D cultures or MRD 3D microtumors compared to clinical samples for genes that were (D) upregulated and (E) downregulated in vitro. (F,G) Dot plots showing pathways enriched among genes uniquely upregulated in MRD 2D compared to clinical samples and related to (F) cell cycle and division, (G) metabolism.

**Figure:**
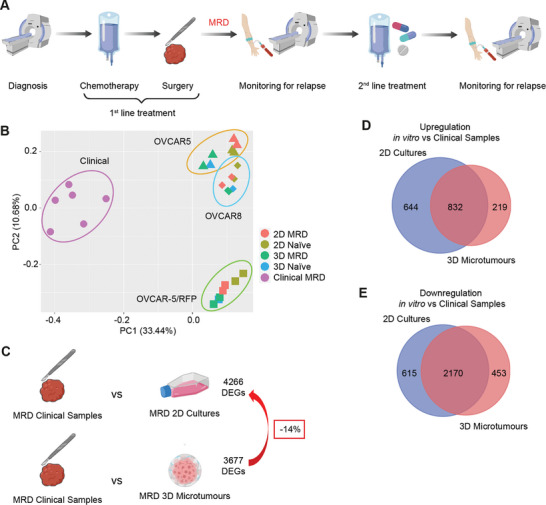

**Figure:**
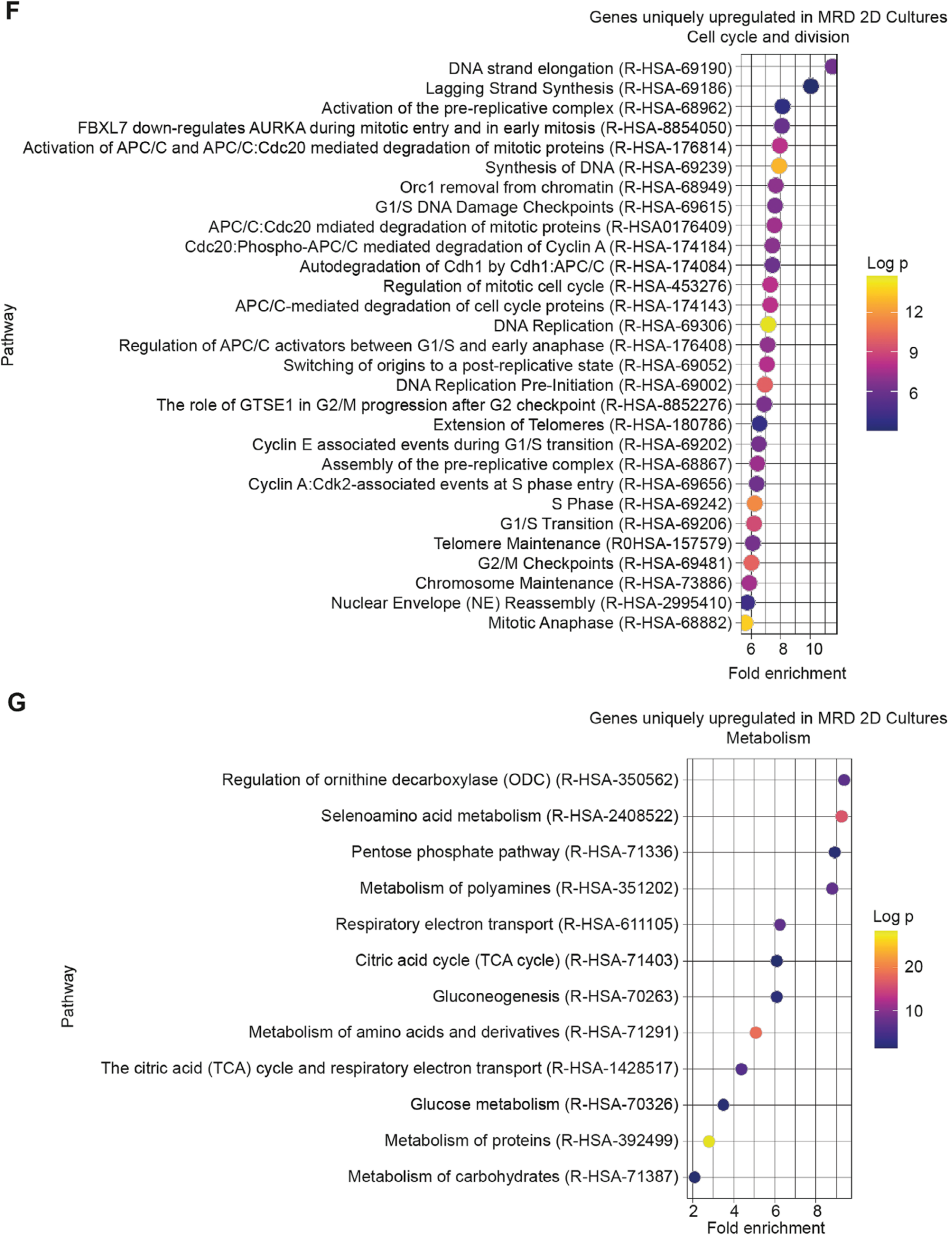

In a previous work, we described how MRD cells show distinctive features such as the upregulation of cancer stem cell markers and genes involved in lipid metabolism, and a more pronounced mesenchymal profile.^[^ ^4^ ^]^ We also developed an MRD 2D in vitro model where treatment‐naïve cancer cells were exposed to carboplatin concentrations to achieve >90% cell killing; the surviving cells recapitulated some of the features of MRD (such as upregulation of lipid metabolism), but lacked the complexity of multicellularity and three‐dimensionality. Therefore, we decided to make microtumors from chemotherapy‐resistant cells and test their suitability as a model for recapitulating MRD biology.

First, we compared the RNA‐Seq data obtained from 2D cultures and 3D microtumors to the previously published libraries obtained from patients with MRD. As shown by Principal Component Analysis (Figure 3B), the major distance is found between the cell lines and the clinical samples, with naïve 2D cells being the furthest away in all three lines.

Also, differential expression analysis identified fewer differentially expressed genes (DEGs) between MRD 3D microtumors and clinical samples than between MRD 2D and clinical samples (Figure 3C). The majority of these DEGs are shared between the two comparisons (Figure 3D,E). If we focus only on the genes uniquely upregulated in MRD 2D, we can appreciate a significant enrichment in pathways related to cell cycle and division (Figure 3F), similar to what we observed when we compared the transcriptomes of naïve 2D cultures and naïve microtumors (Figure S2D, Supporting Information).

**Figure 4:**
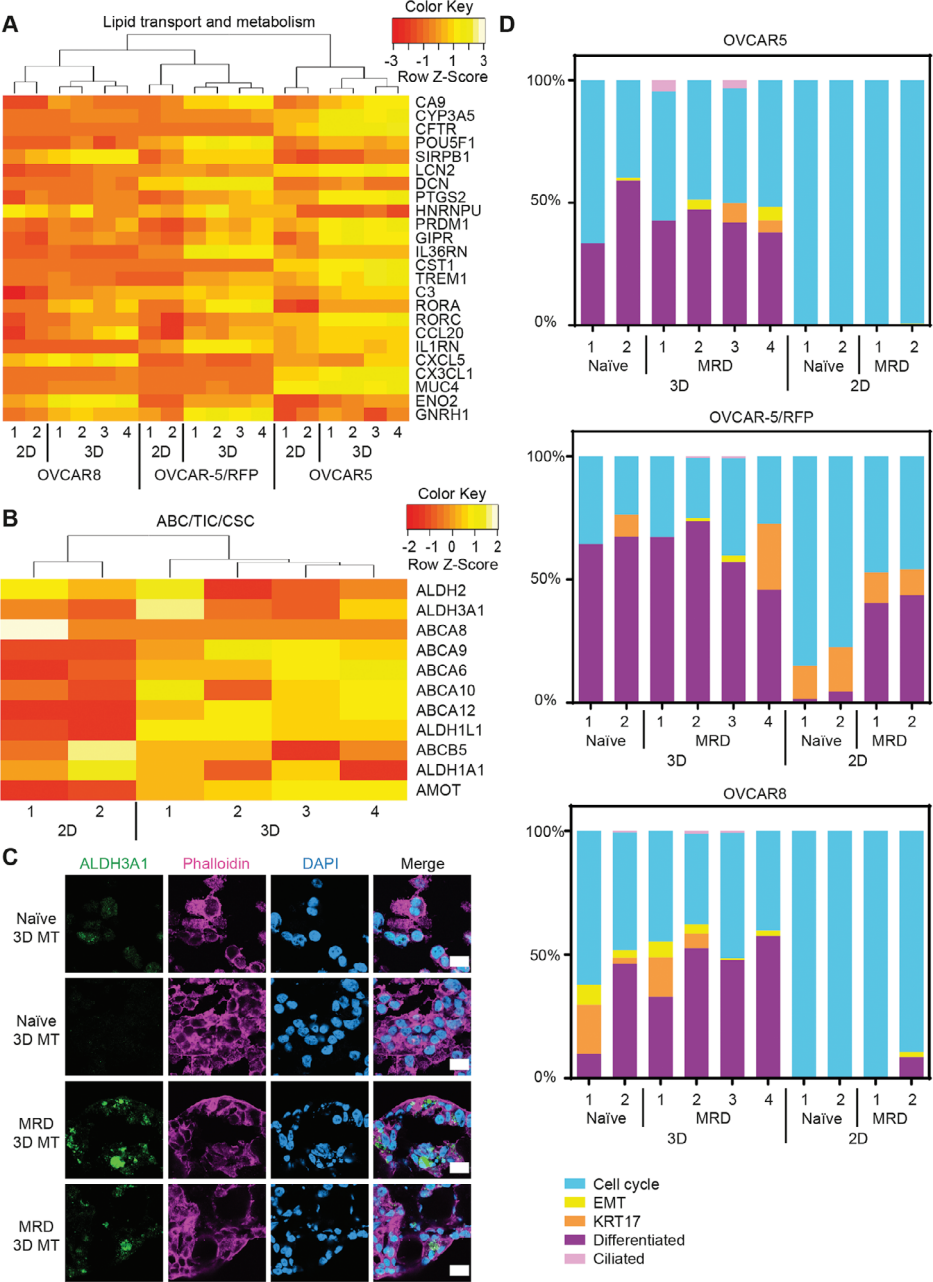
Key features of ovarian cancer MRD recapitulated in 3D microtumors. (A,B) Heatmap showing the expression of genes in MRD 3D microtumors and MRD 2D cultures (A) involved in lipid transport and metabolism, and (B) encoding ATP‐binding cassette (ABC) transporters and markers for tumor‐initiating cells/cancer stem cells (TICs/CSCs). (C) Confocal images of naïve and MRD 3D microtumors (3D MT) at D10 stained with the TIC/CSC marker: ALDH3A1 (green). The cells were also stained with phalloidin (pink), DAPI (blue). Scale bar = 20 µm. (D) Stacked bar plots visualizing the deconvolution result of 3D microtumors and 2D cultures produced from OVCAR5 and OVCAR8. The y‐axis represents the percentage of each cell state in a given sample. Colors of the bars denote the five cell states as shown in the legend.

Other genes exclusively enriched in MRD 2D seem to suggest a different metabolic strategy between these cells and the MRD from clinical samples, with the former based on carbohydrates and amino acids (Figure 3G). This was confirmed by the finding that, when we directly compared the transcriptomes of MRD 2D and MRD 3D microtumors, the latter showed significant upregulation of genes involved in lipid transport and metabolism (**Figure** **4**A), similarly to what we originally found in patients with MRD. Moreover, the expression of genes belonging to the original MRD signature correlated significantly with the expression levels observed in the MRD 3D microtumors (Pearson's correlation coefficient of 0.99, *p*‐value <0.05) (Figure S5A,B, Supporting Information). Importantly, when we compared 3D naïve to 3D MRD cells, the latter still showed an upregulation of lipid‐related genes, demonstrating that this gene signature is not entirely due to the 3D nature of our microtumors and is actually elevated in MRD (Figure S5C, Supporting Information).

Another MRD characteristic that is better recapitulated in 3D microtumors is the increased expression of ATP‐binding cassette (ABC) transporters and markers for tumor‐initiating cells/cancer stem cells (TICs/CSCs): consistent with the fact that MRD lesions survive chemotherapy and are the source of ovarian cancer recurrences, we previously identified an ABC/TIC/CSC gene signature which we now find overexpressed in MRD 3D microtumors compared to MRD 2D (Figure 4B). Additionally, we showed that the aldehyde dehydrogenase ALDH3A1, a known TIC/CSC marker that is also important for fatty acid oxidation (FAO),^[^ ^37^ ^]^ is specifically expressed in the MRD 3D microtumors but not in the naïve 3D microtumors (Figure 4C).

To further explore the nature of pathways and processes that characterize MRD cells in our different models, we also conducted gene set enrichment analyses. This confirmed that several cell cycle and non‐lipid metabolic pathways are more significantly upregulated in 2D cultures (Figure S6, Supporting Information).

Finally, we examined whether microtumors can recapitulate non‐genetic heterogeneity, a key mechanism for the evolution and survival of cancer cells. Tumor heterogeneity is both genetic and non‐genetic, with the latter used to describe cells of the same genetic background but with different phenotypic cell states that can enable invasion, metastasis, and chemotherapy resistance.

We previously reported that ovarian cancer non‐genetic heterogeneity can be measured with molecular signatures related to five different cell states described as the “Oxford Classic” (cell cycle (enriched in cell cycle, DNA repair, and chromatin remodeling pathways), epithelial‐mesenchymal transition (EMT), KRT17 (represented by upregulation of cytokeratins), differentiated (increased in RNA synthesis and transport pathways) and ciliated).^[^ ^38^ ^]^ Deconvolution analysis of our RNA‐Seq dataset showed that all the five gene signatures originally identified in ovarian cancer clinical samples can be found in the 3D microtumors; however, depending on the cell line, only one to three are present in naïve 2D cultures and, consistent with the results we have shown so far, the most abundant is related to the cell cycle state (Figure 4D). We also analyzed a publicly available dataset of 37 additional ovarian cancer cell lines grown in 2D cultures,^[^ ^39^ ^]^ in all of which the cell cycle signature is dominant if not exclusive (Figure S7A, Supporting Information); hence, this is a ubiquitous drawback of monolayer cultures, which fail to recapitulate the essential features of chemo‐resistant cells.

On the other hand, 3D microtumors made from cell lines perform at least as well as organoids established from clinical samples,^[^ ^40^ ^]^ where we observe the occasional sample with only the cell cycle status and very low representation of the ciliated signature (Figure S7B, Supporting Information). Furthermore, in our OVCAR5 and OVCAR‐5/RFP 3D microtumors, the MRD samples show a higher EMT proportion than the naïve cells (Figure 4D); this is again similar to what we observed in our original characterization of MRD clinical samples.^[^ ^4^ ^]^

Taken together, these data provide strong evidence in support of using microtumors to model ovarian cancer MRD; the system successfully recapitulates most of its key features, from lipid metabolism to TICs and EMT.

### 2.4 Using 3D Microtumors as a Drug Testing Platform for Ovarian Cancer MRD

In our previous work, we showed that not only do the MRD cells significantly upregulate their lipid metabolism, but also that this is a vulnerability that can be targeted therapeutically by inhibiting FAO and, in particular, by targeting the carnitine palmitoyl transferase (CPT1) that imports FA into mitochondria for β‐oxidation. This was achieved using our 2D model, where MRD cells treated with the CPT1 inhibitors etomoxir and perhexiline underwent 20–30% more cell death than naïve cells.^[^ ^4^ ^]^

**Figure 5:**
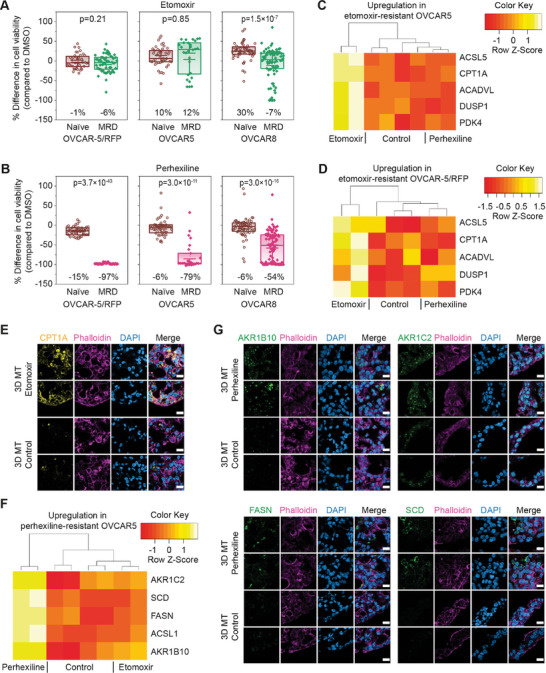
MRD 3D microtumors reveal specific drug responses toward fatty acid oxidation inhibitors. (A,B) Cell viability changes of naïve and MRD 3D microtumors compared to a DMSO control after a 10‐day treatment with (A) 40 µm etomoxir; (B) 4 µm perhexiline (*n* = 25–93 3D microtumors from 3 to 7 independent experiments). (C,D) Heatmaps of differentially expressed genes (DEGs) upregulated in etomoxir‐resistant MRD 3D microtumors composed of (C) OVCAR5 and (D) OVCAR‐5/RFP. (E) Confocal images of MRD 3D microtumors (3D MT) at D10 stained with the FAO marker CPT1A (yellow), phalloidin (pink), DAPI (blue). Scale bar = 20 µm. (F) Heatmap of DEGs upregulated in perhexiline‐resistant MRD 3D microtumors composed of OVCAR5. (G) Confocal images showing the DEGs upregulated in perhexiline‐resistant MRD 3D microtumors composed of OVCAR5 at day 10: AKR1B10 (green, top left panel), AKR1C2 (green, top right panel), FASN (green, bottom left panel), SCD (green, bottom right panel). The cells were also stained with phalloidin (pink), DAPI (blue). Scale bar = 20 µm.

To compare the previous 2D culture data with our 3D model, 3D microtumors were prepared from naïve and MRD cells and treated with etomoxir and perhexiline for a period of 10 days (Figure S8A, Supporting Information). In contrast with the results from 2D cultures, etomoxir failed to induce significant cell death in either naïve or MRD 3D microtumors (**Figure** **5**A), while perhexiline led to a more pronounced reduction in MRD cells in 3D microtumors (Figure 5B) than in 2D cultures (killing MRD cells 48–82% more effectively than naïve cells, Table S8, Supporting Information).

This differential efficacy of FAO inhibitors in 2D cultures versus 3D microtumors could be due to differences in the ability of these compounds to reach the inner cell layers of the 3D microtumors or to their different selectivity (perhexiline inhibits both CPT1 and CPT2 while etomoxir only targets CPT1).^[^ ^41^ ^]^

To gain more insight into this, we analyzed the transcriptomes of the cells in the MRD 3D microtumors that survived the treatment with the inhibitors and compared them to the transcriptomes of the cells in the untreated/DMSO control MRD 3D microtumors. Differential analysis showed that, regardless of the cell line, the etomoxir‐resistant cells upregulated a set of key genes that can increase FAO to different levels (Figure 5C,D; Figure S8B, Supporting Information): beside CPT1 itself, we also found transcripts encoding the long‐chain‐fatty‐acid‐CoA ligase ACSL5, the very long‐chain specific acyl‐CoA dehydrogenase ACADVL, and the pyruvate dehydrogenase kinase PDK4.^[^ ^42^ ^]^ CPT1 and ACADVL upregulation in the etomoxir‐resistant 3D microtumors was also confirmed at protein level through immunofluorescence (Figure 5E; Figure S9, Supporting Information).

The RNA‐Seq analysis of perhexiline‐resistant 3D microtumors showed a different gene signature from those etomoxir‐resistant, even though it was still closely linked to lipid metabolism. Depending on the cell line, the cells that survived perhexiline treatment upregulated at least three to five of the following genes at both the RNA and protein level: the long‐chain‐fatty‐acid‐CoA ligase ACSL1, the fatty acid synthase FASN, the stearoyl‐CoA desaturase SCD and the aldo‐keto reductases AKR1B10 and AKR1C2 (Figure 5F,G; Figure S8C,D, Supporting Information). AKR1B10 has been shown to increase FAO in metastatic breast cancer,^[^ ^43^ ^]^ and the inhibition of AKR1C1/2 can sensitize platinum‐resistant ovarian cancer toward carboplatin.^[^ ^44^ ^]^

Overall, these data confirm once more the potential of the microtumor system, which can be used not only for drug testing purposes but also to investigate resistance mechanisms. More specifically, in this case, our model has enabled us to identify a very promising CPT1/2 inhibitor for targeting ovarian cancer MRD as well as the key genes that we could target simultaneously to avoid the development of resistance.

## 3 Discussion

Drugs targeting MRD would be extremely beneficial for women with ovarian cancer, whose ten‐year survival rate of only 35%^[^ ^45^ ^]^ is much lower than the 54% for all cancers combined.^[^ ^46^ ^]^

In this work, we describe how we have achieved the first 3D model of ovarian cancer MRD using microtumors obtained by microfluidics and we have used them to test potential therapeutics. 3D microtumor models have gained increasing significance over the past decades^[^ ^47^ ^]^ and the FDA Modernization Act 2.0 of December 2022 has eliminated the requirement for animal tests prior to clinical trials, strengthening the position of alternative approaches to drug evaluation.^[^ ^48^ ^]^ This regulatory change signifies a turning away from animal experiments to more effective models for drug evaluation, as over 90% of drugs reaching the bedside have failed due to efficacy and safety issues.^[^ ^49^ ^]^

Here we have shown that our 3D microtumor system represents one such model that satisfies all the technical and biological accuracy requirements to be successfully used in drug testing. From a technical point of view, the most important criteria are uniform size, uniform composition, rapid fabrication, and ease of scalability. The 3D microtumors meet all these requirements, overcoming the weaknesses of conventional spheroid fabrication techniques.

In terms of biological accuracy, our system is able to promptly recapitulate key physiological features observed in vivo: 3D microtumors exhibiting hypoxia can be generated within 1 day, reducing fabrication time by more than 90%^[^ ^24^, ^25^ ^]^; the model also allows an accurate representation of non‐genetic heterogeneity, a crucial player in chemotherapy resistance.^[^ ^50^ ^]^ Our ovarian cancer microtumors displayed all the molecular signatures related to the five different cell states recently described as the “Oxford Classic”,^[^ ^38^, ^51^ ^]^ which is paving the way to patient stratification for this malignancy. Finally, due to their ability to incorporate multiple cell types, the 3D microtumors can recreate the crucial interactions between tumor cells and the microenvironment. This feature cannot be easily recapitulated in otherwise powerful 3D cultures, like patient‐derived organoids, and represents one of their main disadvantages.^[^ ^52^ ^]^ As an example, we were able to use our model to co‐culture ovarian cancer cells and fibroblasts.

Transcriptomics analysis showed that our 3D microtumors are a reliable model for ovarian cancer MRD, with the main aspects of MRD biology related to lipid metabolism and TICs successfully recapitulated.

We used the 3D microtumors to test two inhibitors of FAO, which were previously found to selectively kill MRD cells in a 2D format, but to be ineffective toward naïve ovarian cancer cells.^[^ ^4^ ^]^ When applied to 3D microtumors, etomoxir showed no cell killing while perhexiline had a far greater cytotoxicity toward MRD cells in the 3D than the 2D format. Importantly, perhexiline is currently used in the clinic as a prophylactic antianginal agent and the doses that elicited a response in our system could potentially be delivered safely and locally in ovarian cancer patients using Hyperthermic Intraperitoneal Chemotherapy. Moreover, based on our transcriptomic analysis of the very few cells surviving perhexiline treatment, this population could potentially be eliminated using aldose reductase inhibitors, some of which have successfully been used to reverse drug resistance in prostate and colorectal cancer lines.^[^ ^53^ ^]^

## 4 Conclusion

In this study, we have developed an in vitro 3D model that recapitulates the characteristics of clinical MRD. Our findings could lead us a step closer to personalized medicine in the treatment of ovarian cancer. Large interpatient variability is observed in cancer pharmacology and is driven by polymorphisms as well as each tumor's genetic and non‐genetic heterogeneity, which our system can successfully recreate. Therefore, we can imagine a future scenario where biopsies are collected during the diagnostic laparoscopy or following neoadjuvant chemotherapy and then used to create 3D microtumors for the testing of different lipid metabolism inhibitors as well as resistance mechanisms. Each patient would then receive the drug combination that proved to be the most efficient at killing her tumor cells ex vivo.

Importantly, this work also represents a crucial proof of concept for the use of 3D microtumors produced by microfluidics as a drug testing platform and, given its versatility, the system could potentially be used to fabricate 3D models of several different types of tumors.

## 5 Experimental Section

### Cell Culture

OVCAR‐5/RFP, NIH3T3/GFP, MDA‐MB‐231/RFP, and HeLa/GFP cell lines were purchased from Cell Biolabs Inc., USA. HEK293T and 3T3‐L1 cell lines were purchased from ATCC. Kuramochi cells were obtained from the JCRB Cell Bank. All cells except 3T3‐L1 were cultured in DMEM (Sigma–Aldrich, #D5796), supplemented with 10% (v/v) FBS (Sigma–Aldrich, #F7524), 2 mm GlutaMAX Supplement (Gibco, #35050038), 0.1 mm MEM NEAA (Sigma–Aldrich, #M7145), and 1% (v/v) Penicillin‐Streptomycin (Pen‐Strep, 100 U mL^−1^ and 100 µg mL^−1^ respectively, Sigma–Aldrich, #P4333). 3T3‐L1 cells were cultured routinely in DMEM supplemented with 10% (v/v) bovine calf serum (ATCC, #30‐2020) and 1% (v/v) Pen‐Strep. To induce differentiation of 3T3‐L1 cells into adipocytes, the cells were cultured in differentiation medium following an established protocol (Table S9, Supporting Information).^[^ ^54^ ^]^

### Cell‐Hydrogel Suspension Preparation

Matrigel Matrix (#354234) and Collagen I (#354236) were purchased from Corning Life Sciences, UK. The gels were thawed completely on ice before use. Collagen I solution (2 mg mL^−1^) was prepared by diluting Collagen I (3.78 mg mL^−1^, 52.9 µL) with ice‐cold DI‐water (39.5 µL), 10X DPBS (6.15 µL) and 1 N NaOH (1.2 µL). Agarose solution (2% w/v) was prepared by dissolving agarose powder (Thermo Fisher, #16520050) in sterile water at 100 °C, then cooled to 37 °C. Silk fibroin solution (50 mg mL^−1^, Sigma–Aldrich, #5154) was thawed at 4 °C and supplemented with 10 U mL^−1^ horseradish peroxidase (type VI lyophilized powder, Sigma‐Aldrich) and 0.4 µL mL^−1^ hydrogen peroxide solution (30% w/w, Sigma Aldrich). The cell‐hydrogel suspension, with cell density = 3–4 × 10^7^ cells mL^−1^, was prepared by resuspending cell pellets in the desired pre‐gel solution (Table S1, Supporting Information).

### Microfluidics Platform and 3D Microtumor Fabrication

The microfluidics platform was improved over the work previously reported by our group.^[^ ^55^ ^]^ The PDMS microfluidics chips (Figure S1A, Supporting Information) were prepared by casting on a custom‐made reverse mold, which was produced by a 3D printer (Solid Print3D, Formlabs) using clear resin (Formlabs), and provided more choices of channel size compared to the previous method of drilling holes in PDMS blocks to make the T‐junction. Large 3D microtumors (size ≈900 µm) in this work were created with microfluidics chips prepared with the drilling hole method, while medium and small 3D microtumors (≈650 and 300 µm in size) were created using microfluidics chips prepared with the 3D printed reverse mold method. The cell‐hydrogel suspension and the oil, tetradecane (Sigma–Aldrich, #172456), were loaded into separate syringes (Figure 1Ai), and pumped into the three‐channel microfluidics chip with neMESYS syringe pumps (Cetoni, Korbussen, Germany). Droplets containing cells in Matrigel, separated by the oil, were formed in a PTFE exit tube (Cole‐Parmer, UK). Upon complete gelation (Table S2, Supporting Information), the 3D microtumors were ejected from the exit tube, transferred to cell culture medium, and maintained at 37 °C, 5% CO_2_. Co‐culture 3D microtumors in this work are composed of a 50:50 mixture of OVCAR‐5/RFP tumor cells and 3T3 fibroblasts.

### Characterizations of 3D Microtumors—Size Distribution

The 3D microtumors were imaged by using a Leica DMi8 inverted epifluorescence microscope platform equipped with a Leica DFC7000 CCD camera (Leica Microsystems Ltd, UK). Images were processed with Fiji ImageJ software to obtain the diameter of each 3D microtumor. For 3D microtumors with the cross‐section of an ellipse, the dimensions were defined as $size=\sqrt{Major\;axis\times Minor\;axis}$.

### Characterizations of 3D Microtumors—Cell Viability

The viabilities of cells in 2D culture and cell‐hydrogel suspension were determined with a Countess Automated Cell Counter (Invitrogen) by using 0.4% trypan blue solution (Invitrogen, #C10314).

The viabilities of cells in 3D microtumors were evaluated with PrestoBlue Cell Viability Reagent (Thermo Fisher, #A13261) according to the manufacturer's instructions. Fluorescence intensity was measured with a microplate reader (CLARIOstar Plus, BMG LABTECH) (Table S10, Supporting Information).

### Characterizations of 3D Microtumors—Hypoxia Staining

Image‐iT Green Hypoxia Reagent (Thermo Fisher, #I14834) was dissolved in DMSO (Sigma–Aldrich, #D8418) to prepare a 5 mm stock solution, which was added to culture medium at a final concentration of 5 µm. After incubation at 37 °C for 3 h, 3D microtumors were imaged with a confocal laser scanning microscope (Leica TCS SP5, Leica Microsystems). A standard FITC/GFP excitation/emission filter set was applied. Z‐stack images were taken with an optical section thickness of 10 µm. The optical z slices were projected to form 2D images using z‐project in Fiji ImageJ software.

### Anticancer Drug Responses

Carboplatin powder (Cayman Chemical, #13112) was dissolved and then serially diluted with sterile water to yield a range of working solutions, which were added to cell medium at a 1:20 volume ratio to a maximum final concentration of 500 µm.

Paclitaxel powder (Invitrogen, #P3456) was dissolved and then serially diluted with DMSO to yield a range of working solutions, which were added to cell medium at a 1:100 volume ratio to a maximum final concentration of 1 µm.

For drug treatment, one microtumor or 5000 cells (2D) was seeded into each well of a 96‐well plate (Corning #3595). After 2 d, the medium was replaced with drug‐containing medium, and the treatment was continued for 4 d. PrestoBlue was used to evaluate cell viability at the end of the drug treatment. Sample size for each condition, 2D cells *n* = 11–21, 3D microtumors *n* = 20–32.

OriginPro 2023 (OriginLab Corporation) was used to plot cell viability data and generate fitted dose‐response curves. The IC_50_ values were derived from the dose‐response curves at 50% cell viability.

### Minimal Residual Disease Modeling—Preparation of MRD‐Like Cells

OVCAR5 and OVCAR8 cell lines were obtained from ATCC and cultured in RPMI 1640 (Gibco, Thermo Fisher, #21875034), supplemented with 10% (v/v) FBS and 1% (v/v) Pen‐Strep.

To prepare MRD‐like cells, 2D naïve cancer cells were treated with carboplatin for 2 weeks under conditions optimized to achieve more than 90% cell killing as previously described^[^ ^4^ ^]^ (5 µg mL^−1^ for KURAMOCHI, 3 µg mL^−1^ for OVCAR5, 2 µg mL^−1^ for OVCAR8). All the cells collected on D14 were expanded for 2–14 days (depending on cell growth) to produce the MRD cells for later use.

### Minimal Residual Disease Modeling—Fatty Acid Oxidation Inhibitor Responses

Etomoxir sodium salt (Stratech, #S8244‐SEL) was dissolved in DMSO to prepare a 40 mm stock solution, which was diluted in cell medium to a final concentration of 40 µm. Perhexiline (Cambridge Bioscience, #CAY16982) was dissolved in DMSO to prepare a 4 mm perhexiline stock solution, which was diluted in cell medium to a final concentration of 4 µm. DMSO was added to cell medium at a 1:1000 volume ratio for the control group.

3D microtumors were prepared from both naïve and MRD cells. Five 3D microtumors were seeded into each well of a 12‐well plate on the day of fabrication and cultured with 2 mL of drug‐containing or DMSO‐containing medium for 10 d. PrestoBlue was used to evaluate cell viability at the end of the drug treatment.

### Minimal Residual Disease Modeling—RNA Extraction and Library Preparation

RNA was extracted with the RNAqueous‐Micro Total RNA Isolation Kit (Thermo Fisher, #AM1931). RNA integrity was evaluated by RIN value with the 2200 TapeStation System (Agilent Technologies, Inc.) and only samples with RIN values above seven were taken forward for library preparation, which was performed using a KAPA HyperPrep Kit (Kapa Biosystems, #KR1351) following the manufacturer's instructions. The libraries were evaluated by using the 2200 TapeStation System and then quantified with a Qubit 2.0 Fluorometer (Thermo Fisher, Invitrogen). Multiplexed library pools of different samples were quantified with the KAPA Library Quantification Kit (Roche) and sequenced by using 75 bp paired‐end reads on the NextSeq500 platform (Illumina).

### Minimal Residual Disease Modeling—Processing of RNA‐Seq data

Sequencing reads from FASTQ files were trimmed for adapter sequences and quality with Trim Galore!, and mapped to the UCSC hg19 human genome assembly using STAR (v2.7.3a). Read counts were obtained by using subread FeatureCounts (v2.0.0).

Differential expression analysis was carried out by using edgeR (v3.36.0). Statistical overrepresentation analysis was performed with PANTHER (v17), and the threshold for significance was set at FDR < 0.05.

Deconvolution analysis was performed as previously described^[^ ^38^ ^]^ in the relative mode, and thus, for each tumor the scores of the five molecular signatures added up to 1.

### Minimal Residual Disease Modeling—Study approval

The HGSOC clinical samples used in this study were recruited under the Gynaecological Oncology Targeted Therapy Study 01 (GO‐Target‐01, NHS Health Research Authority South Central – Berkshire Research Ethics Committee research ethics approval 11‐SC‐0014) and the Oxford Ovarian Cancer Predict Chemotherapy Response Trial (OXO‐PCR‐01, NHS Health Research Authority South Central – Berkshire Research Ethics Committee research ethics approval 12‐SC‐0404). All participants involved in this study were appropriately informed and consented.

### Minimal Residual Disease Modeling—Immunofluorescence Staining

The 3D microtumors and the clinical samples were embedded in OCT (NEG‐50, Richard‐Allan Scientific), frozen, and kept at −80 °C until sectioning. 10 µm sections were taken in a CryoStar cryostat microtome (Thermo Fisher) and stained for immunofluorescence imaging. The slides were washed with ice‐cold PBS twice to remove the OCT, fixed in 4% PFA for 10 min and permeabilized with 0.1%TritonX‐100 in PBS for 10 min at RT. The samples were then incubated in Blocking Buffer (2% BSA + 0.1% TritonX‐100 in PBS) for 1 h followed by an overnight incubation with the diluted primary antibodies (Table S11, Supporting Information) in a humidified chamber at 4 °C. The following day the slides were washed in PBS and incubated with the secondary antibodies and phalloidin for 1 h at RT. After extensive washes in PBS, the slides were mounted with Vectashield + DAPI (VectorLaboratories) and dried in the dark before being imaged using a confocal microscope (Zeiss900).

### Statistical Analysis

Data were analyzed using Origin 2023 software (OriginLab Corporation). Statistical significance was tested using a Tukey test and is indicated by *p < 0.05, **p < 0.01, and ***p < 0.001. Sample size (*n*) for each statistical analysis is indicated in figure legends. Data are expressed as mean ± standard deviation of the mean (SD).

## Conflict of Interest

The authors declare no conflict of interest.

## Author Contributions

X.Y. and M.A. contributed equally to this work. X.Y., M.A., H.B., and A.A.A. conceived and designed the work, which was supervised by H.B. and A.A.A. X.Y. improved the microfluidics platform, and performed 3D microtumor fabrication and chemotherapy assays. X.Y. and M.A. contributed to cell culture and manipulation, microscope imaging, cryo‐sectioning, RNA‐Seq library preparation, and data and image analysis. M.A. performed the bioinformatics analysis and immunofluorescence. L.R. performed the deconvolution analysis. Y.J. and L.Z. contributed to cryo‐sectioning and cell staining. Y.Z. contributed to the preparation of silk fibroin and reverse molds of microfluidics chips. E.M. contributed to cell monolayer culture. N.M. and S.M. assisted with preliminary experiments. R.K.K., S.M.‐G., A.Aggarwal, and L.Z. contributed discussions and helped with image analysis. A.Albhuari contributed discussions. X.Y., M.A., A.Aggarwal, H.B., and A.A.A. wrote the manuscript. H.B., A.A.A., L.Z., and M.A. acquired funding. All authors read and revised the manuscript.

## Supporting information



Supporting Information

## Data Availability

The data that support the findings of this study are available from the corresponding author upon reasonable request.

## References

[1] A. Ramos , S. Sadeghi , H. Tabatabaeian , Int. J. Mol. Sci. 2021, 22, 9451. 34502361 10.3390/ijms22179451 PMC8430957

[2] M. R. Luskin , M. A. Murakami , S. R. Manalis , D. M. Weinstock , Nat. Rev. Cancer 2018, 18, 255. 29376520 10.1038/nrc.2017.125 PMC6398166

[3] T. G. Bivona , R. C. Doebele , Nat. Med. 2016, 22, 472. 27149220 10.1038/nm.4091 PMC5384713

[4] M. Artibani , K. Masuda , Z. Hu , P. C. Rauher , G. Mallett , N. Wietek , M. Morotti , K. Chong , M. KaramiNejadRanjbar , C. E. Zois , S. Dhar , S. El‐Sahhar , L. Campo , S. P. Blagden , S. Damato , P. N. Pathiraja , S. Nicum , F. Gleeson , A. Laios , A. Alsaadi , L. Santana Gonzalez , T. Motohara , A. Albukhari , Z. Lu , R. C. Bast Jr. , A. L. Harris , C. S. Ejsing , R. W. Klemm , C. Yau , T. Sauka‐Spengler , et al., JCI Insight 2021, 6, e147929. 33945502 10.1172/jci.insight.147929 PMC8262282

[5] P. A. Johnson , J. R. Giles , Nat. Rev. Cancer 2013, 13, 432. 23676850 10.1038/nrc3535

[6] W. Asghar , R. El Assal , H. Shafiee , S. Pitteri , R. Paulmurugan , U. Demirci , Mater. Today 2015, 18, 539. 10.1016/j.mattod.2015.05.002 PMC5407188 28458612

[7] K. Duval , H. Grover , L.‐H. Han , Y. Mou , A. F. Pegoraro , J. Fredberg , Z. Chen , Physiology 2017, 32, 266. 28615311 10.1152/physiol.00036.2016 PMC5545611

[8] T. Velletri , C. E. Villa , D. Cilli , B. Barzaghi , P. Lo Riso , M. Lupia , R. Luongo , A. Lopez‐Tobon , M. De Simone , R. J. P. Bonnal , L. Marelli , S. Piccolo , N. Colombo , M. Pagani , U. Cavallaro , S. Minucci , G. Testa , Cell Death Differ. 2022, 29, 614. 34845371 10.1038/s41418-021-00878-w PMC8901794

[9] C. Jensen , Y. Teng , Front. Mol. Biosci. 2020, 7, 33. 32211418 10.3389/fmolb.2020.00033 PMC7067892

[10] a) M. Kapałczyńska , T. Kolenda , W. Przybyła , M. Zajączkowska , A. Teresiak , V. Filas , M. Ibbs , R. Bliźniak , Ł. Łuczewski , K. Lamperska , Arch. Med. Sci. 2018, 14, 910; 30002710 10.5114/aoms.2016.63743 PMC6040128

[11] K. O. Rojek , M. Ćwiklińska , J. Kuczak , J. Guzowski , Chem. Rev. 2022, 122, 16839. 36108106 10.1021/acs.chemrev.1c00798 PMC9706502

[12] M. B. Meads , R. A. Gatenby , W. S. Dalton , Nat. Rev. Cancer 2009, 9, 665. 19693095 10.1038/nrc2714

[13] a) Z. Koledova , 3D Cell Culture: Methods and Protocols, (Ed: Z. Koledova ), Springer, New York 2017;

[14] a) F. Foglietta , R. Canaparo , G. Muccioli , E. Terreno , L. Serpe , Life Sci. 2020, 254, 117784; 32416169 10.1016/j.lfs.2020.117784

[15] C. Zuppinger , Front. Cardiovasc. Med. 2019, 6, 87. 31294032 10.3389/fcvm.2019.00087 PMC6606697

[16] S. Breslin , L. O'Driscoll , Drug Discovery Today 2013, 18, 240. 23073387 10.1016/j.drudis.2012.10.003

[17] E. Knight , S. Przyborski , J. Anat. 2015, 227, 746. 25411113 10.1111/joa.12257 PMC4694114

[18] S. Jiang , H. Zhao , W. Zhang , J. Wang , Y. Liu , Y. Cao , H. Zheng , Z. Hu , S. Wang , Y. Zhu , W. Wang , S. Cui , P. E. Lobie , L. Huang , S. Ma , Cell Rep. Med. 2020, 1, 100161. 33377132 10.1016/j.xcrm.2020.100161 PMC7762778

[19] Z. Hu , Y. Cao , E. A. Galan , L. Hao , H. Zhao , J. Tang , G. Sang , H. Wang , B. Xu , S. Ma , ACS Biomater. Sci. Eng. 2022, 8, 1215. 35167260 10.1021/acsbiomaterials.1c01099

[20] Y. Cao , J. Tan , H. Zhao , T. Deng , Y. Hu , J. Zeng , J. Li , Y. Cheng , J. Tang , Z. Hu , K. Hu , B. Xu , Z. Wang , Y. Wu , P. E. Lobie , S. Ma , Nat. Commun. 2022, 13, 7463. 36460667 10.1038/s41467-022-35183-8 PMC9718784

[21] S. Ding , C. Hsu , Z. Wang , N. R. Natesh , R. Millen , M. Negrete , N. Giroux , G. O. Rivera , A. Dohlman , S. Bose , T. Rotstein , K. Spiller , A. Yeung , Z. Sun , C. Jiang , R. Xi , B. Wilkin , P. M. Randon , I. Williamson , D. A. Nelson , D. Delubac , S. Oh , G. Rupprecht , J. Isaacs , J. Jia , C. Chen , J. P. Shen , S. Kopetz , S. McCall , A. Smith , et al., Cell Stem Cell 2022, 29, 905. 35508177 10.1016/j.stem.2022.04.006 PMC9177814

[22] R. K. Kumar , T. A. Meiller‐Legrand , A. Alcinesio , D. Gonzalez , D. A. I. Mavridou , O. J. Meacock , W. P. J. Smith , L. Zhou , W. Kim , G. S. Pulcu , H. Bayley , K. R. Foster , Nat. Commun. 2021, 12, 857. 33558498 10.1038/s41467-021-20996-w PMC7870943

[23] A. Klemba , L. Bodnar , H. Was , K. K. Brodaczewska , G. Wcislo , C. A. Szczylik , C. Kieda , Int. J. Mol. Sci. 2020, 21, 9492. 33327450 10.3390/ijms21249492 PMC7764929

[24] a) S. Riffle , R. S. Hegde , J. Exp. Clin. Cancer Res. 2017, 36, 102; 28774341 10.1186/s13046-017-0570-9 PMC5543535

[25] a) A. A. Popova , T. Tronser , K. Demir , P. Haitz , K. Kuodyte , V. Starkuviene , P. Wajda , P. A. Levkin , Small 2019, 15, 1901299; 10.1002/smll.201901299 31058427

[26] A. F. Baker , S. W. Malm , R. Pandey , C. Laughren , H. Cui , D. Roe , S. K. Chambers , Cancer Microenviron. 2015, 8, 45. 25998313 10.1007/s12307-015-0166-x PMC4449346

[27] R. P. Stevenson , D. Veltman , L. M. Machesky , J. Cell Sci. 2012, 125, 1073. 22492983 10.1242/jcs.093799

[28] M. F. Olson , E. Sahai , Clin. Exp. Metastasis 2009, 26, 273. 18498004 10.1007/s10585-008-9174-2

[29] N. V. Klementieva , L. B. Snopova , N. N. Prodanets , O. E. Furman , V. V. Dudenkova , E. V. Zagaynova , K. A. Lukyanov , A. S. Mishin , Anticancer Res. 2016, 36, 5287. 27798890 10.21873/anticanres.11100

[30] S. Oguri , T. Sakakibara , H. Mase , T. Shimizu , K. Ishikawa , K. Kimura , R. D. Smyth , J. Clin. Pharmacol. 1988, 28, 208. 3283185 10.1002/j.1552-4604.1988.tb03134.x

[31] R. Kessous , M. D. Wissing , S. Piedimonte , J. Abitbol , L. Kogan , I. Laskov , A. Yasmeen , S. Salvador , S. Lau , W. H. Gotlieb , Acta Obstet. Gynecol. Scand. 2020, 99, 933. 31954071 10.1111/aogs.13814

[32] T. B. Stage , T. K. Bergmann , D. L. Kroetz , Clin. Pharmacokinet. 2018, 57, 7. 28612269 10.1007/s40262-017-0563-z PMC8572663

[33] M. Zhang , Z. Chen , Y. Wang , H. Zhao , Y. Du , Cancers (Basel) 2022, 14, 2637. 35681617 10.3390/cancers14112637 PMC9179444

[34] C. Angelucci , G. Maulucci , G. Lama , G. Proietti , A. Colabianchi , M. Papi , A. Maiorana , M. De Spirito , A. Micera , O. B. Balzamino , A. Di Leone , R. Masetti , G. Sica , PLoS One 2012, 7, e50804. 23251387 10.1371/journal.pone.0050804 PMC3519494

[35] Y. L. Huang , C. Shiau , C. Wu , J. E. Segall , M. Wu , Biophys. Rev. Lett. 2020, 15, 131. 33033500 10.1142/s1793048020500034 PMC7540657

[36] R. Li , D. Zhang , B. Ren , S. Cao , L. Zhou , Y. Xiong , Q. Sun , X. Ren , Bull. Cancer 2023, 110, 285. 36739242 10.1016/j.bulcan.2022.11.013

[37] J. S. Lee , S. H. Kim , S. Lee , J. H. Kang , S. H. Lee , J. H. Cheong , S. Y. Kim , Sci. Rep. 2019, 9, 16313. 31705020 10.1038/s41598-019-52814-1 PMC6841934

[38] Z. Hu , M. Artibani , A. Alsaadi , N. Wietek , M. Morotti , T. Shi , Z. Zhong , L. Santana Gonzalez , S. El‐Sahhar , M. KaramiNejadRanjbar , G. Mallett , Y. Feng , K. Masuda , Y. Zheng , K. Chong , S. Damato , S. Dhar , L. Campo , R. Garruto Campanile , H. Soleymani majd , V. Rai , D. Maldonado‐Perez , S. Jones , V. Cerundolo , T. Sauka‐Spengler , C. Yau , A. A. Ahmed , Cancer Cell 2020, 37, 226. 32049047 10.1016/j.ccell.2020.01.003

[39] T. A. Ince , A. D. Sousa , M. A. Jones , J. C. Harrell , E. S. Agoston , M. Krohn , L. M. Selfors , W. Liu , K. Chen , M. Yong , P. Buchwald , B. Wang , K. S. Hale , E. Cohick , P. Sergent , A. Witt , Z. Kozhekbaeva , S. Gao , A. T. Agoston , M. A. Merritt , R. Foster , B. R. Rueda , C. P. Crum , J. S. Brugge , G. B. Mills , Nat. Commun. 2015, 6, 7419. 26080861 10.1038/ncomms8419 PMC4473807

[40] O. Kopper , C. J. de Witte , K. Lohmussaar , J. E. Valle‐Inclan , N. Hami , L. Kester , A. V. Balgobind , J. Korving , N. Proost , H. Begthel , L. M. van Wijk , S. A. Revilla , R. Theeuwsen , M. van de Ven , M. J. van Roosmalen , B. Ponsioen , V. W. H. Ho , B. G. Neel , T. Bosse , K. N. Gaarenstroom , H. Vrieling , M. P. G. Vreeswijk , P. J. van Diest , P. O. Witteveen , T. Jonges , J. L. Bos , A. van Oudenaarden , R. P. Zweemer , H. J. G. Snippert , W. P. Kloosterman , et al., Nat. Med. 2019, 25, 838. 31011202 10.1038/s41591-019-0422-6

[41] S. Inglis , S. Stewart , Eur. J. Cardiovasc. Nurs. 2006, 5, 175. 16469541 10.1016/j.ejcnurse.2006.01.001

[42] I. K. N. Pettersen , D. Tusubira , H. Ashrafi , S. E. Dyrstad , L. Hansen , X. Z. Liu , L. I. H. Nilsson , N. G. Lovsletten , K. Berge , H. Wergedahl , B. Bjorndal , O. Fluge , O. Bruland , A. C. Rustan , N. Halberg , G. V. Rosland , R. K. Berge , K. J. Tronstad , Mitochondrion 2019, 49, 97. 31351920 10.1016/j.mito.2019.07.009

[43] A. van Weverwijk , N. Koundouros , M. Iravani , M. Ashenden , Q. Gao , G. Poulogiannis , U. Jungwirth , C. M. Isacke , Nat. Commun. 2019, 10, 2698. 31221959 10.1038/s41467-019-10592-4 PMC6586667

[44] S. Badmann , D. Mayr , E. Schmoeckel , A. Hester , C. Buschmann , S. Beyer , T. Kolben , F. Kraus , A. Chelariu‐Raicu , A. Burges , S. Mahner , U. Jeschke , F. Trillsch , B. Czogalla , Sci. Rep. 2022, 12, 1862. 35115586 10.1038/s41598-022-05785-9 PMC8814148

[45] CRUK , Ovarian cancer survival statistics, https://www.cancerresearchuk.org/health‐professional/cancer‐statistics/statistics‐by‐cancer‐type/ovarian‐cancer/survival#heading‐Zero, (accessed: March 2023).

[46] CRUK , Cancer survival statistics for all cancers combined, https://www.cancerresearchuk.org/health‐professional/cancer‐statistics/survival/all‐cancers‐combined#heading‐Zero, (accessed: March 2023).

[47] L. P. Ferreira , V. M. Gaspar , J. F. Mano , Acta Biomater. 2018, 75, 11. 29803007 10.1016/j.actbio.2018.05.034 PMC7617007

[48] M. Wadman , Science 2023, 379, 127. 36634170 10.1126/science.adg6276

[49] a) A. A. Seyhan , Transl. Med. Commun. 2019, 4, 18;

[50] J. C. Marine , S. J. Dawson , M. A. Dawson , Nat. Rev. Cancer 2020, 20, 743. 33033407 10.1038/s41568-020-00302-4

[51] Z. Hu , P. Cunnea , Z. Zhong , H. Lu , O. I. Osagie , L. Campo , M. Artibani , K. Nixon , J. Ploski , L. Santana Gonzalez , A. Alsaadi , N. Wietek , S. Damato , S. Dhar , S. P. Blagden , C. Yau , J. Hester , A. Albukhari , E. O. Aboagye , C. Fotopoulou , A. Ahmed , Clin. Cancer Res. 2021, 27, 1570. 33446563 10.1158/1078-0432.CCR-20-2782

[52] V. Veninga , E. E. Voest , Cancer Cell 2021, 39, 1190. 34416168 10.1016/j.ccell.2021.07.020

[53] T. M. Penning , S. Jonnalagadda , P. C. Trippier , T. L. Rizner , Pharmacol. Rev. 2021, 73, 1150. 34312303 10.1124/pharmrev.120.000122 PMC8318518

[54] C. A. C. Freyre , P. C. Rauher , C. S. Ejsing , R. W. Klemm , Mol. Cell 2019, 76, 811. 31628041 10.1016/j.molcel.2019.09.011

[55] S. Ma , N. Mukherjee , E. Mikhailova , H. Bayley , Adv. Biosyst. 2017, 1, 1700075. 10.1002/adbi.201700075 32646178

