# Supplementary material for: 3D Microtumors Representing Ovarian Cancer Minimal Residual Disease Respond to the Fatty Acid Oxidation Inhibitor Perhexiline
Source: Adv Healthc Mater. 2025 Feb 9;14(14):2404072. doi: 10.1002/adhm.202404072 (PMC12118330; doi:10.1002/adhm.202404072)
Supplement: Supplementary file 1 — Supporting Information [file ADHM-14-0-s001.pdf]

ADVANCED HEALTHCARE MATERIALS

Supporting Information

for *Adv. Healthcare Mater.*, DOI 10.1002/adhm.202404072

3D Microtumors Representing Ovarian Cancer Minimal Residual Disease Respond to the Fatty Acid Oxidation Inhibitor Perhexiline

Xingyun Yang, Mara Artibani, Yongcheng Jin, Aneesh Aggarwal, Yujia Zhang, Sandra Muñoz-Galvan, Ellina Mikhailova, Lena Rai, Nobina Mukherjee, Ravinash Krishna Kumar, Ashwag Albukhari, Shaohua Ma, Linna Zhou, Ahmed Ashour Ahmed* and Hagan Bayley**

Supporting Information

3D microtumors representing ovarian cancer minimal residual disease respond to the fatty acid oxidation inhibitor perhexiline

Xingyun Yang, Mara Artibani, Yongcheng Jin, Aneesh Aggarwal, Yujia Zhang, Sandra Muñoz-Galvan, Ellina Mikhailova, Lena Rai, Nobina Mukherjee, Ravinash Krishna Kumar, Ashwag Albukhari, Shaohua Ma, Linna Zhou, Ahmed Ashour Ahmed*, Hagan Bayley**

Affiliation

X. Yang, Y. Jin, Y. Zhang, E. Mikhailova, N. Mukherjee, R. K. Kumar, L. Zhou, H. Bayley

Department of Chemistry

University of Oxford

Oxford, OX1 3TA, UK

Email: linna.zhou@ludwig.ox.ac.uk; hagan.bayley@chem.ox.ac.uk

M. Artibani, A. Aggarwal, S. Muñoz-Galvan, L. Rai, A. A. Ahmed

Ovarian Cancer Cell Laboratory, MRC Weatherall Institute of Molecular Medicine

University of Oxford

Oxford, OX3 9DS, UK

Email: ahmed.ahmed@wrh.ox.ac.uk

M. Artibani, A. Aggarwal, L. Rai, A. A. Ahmed

Nuffield Department of Women's & Reproductive Health

University of Oxford

Oxford, OX3 9DU, UK

Y. Zhang

Institute of Electrical and Microengineering

École Polytechnique Fédérale de Lausanne

Lausanne, 1015, Switzerland

S. Muñoz-Galvan

Instituto de Biomedicina de Sevilla

IBiS/Hospital Universitario Virgen del Rocío/CSIC/Universidad de Sevilla, Avda

Manuel Siurot s/n 41013, Seville, Spain

R. K. Kumar

Department of Infectious Disease

Imperial College London

South Kensington, London, SW7 2AZ, UK

A. Albukhari

Biochemistry Department, Faculty of Science

King Abdulaziz University

Jeddah, Saudi Arabia

S. Ma

Tsinghua Shenzhen International Graduate School (SIGS)

Tsinghua University

Shenzhen, 518055, China

S. Ma

Tsinghua-Berkeley Shenzhen Institute (TBSI)

Tsinghua University

Shenzhen, 518055, China

L. Zhou

Ludwig Institute for Cancer Research, Nuffield Department of Medicine

University of Oxford

Oxford, OX3 7DQ, UK

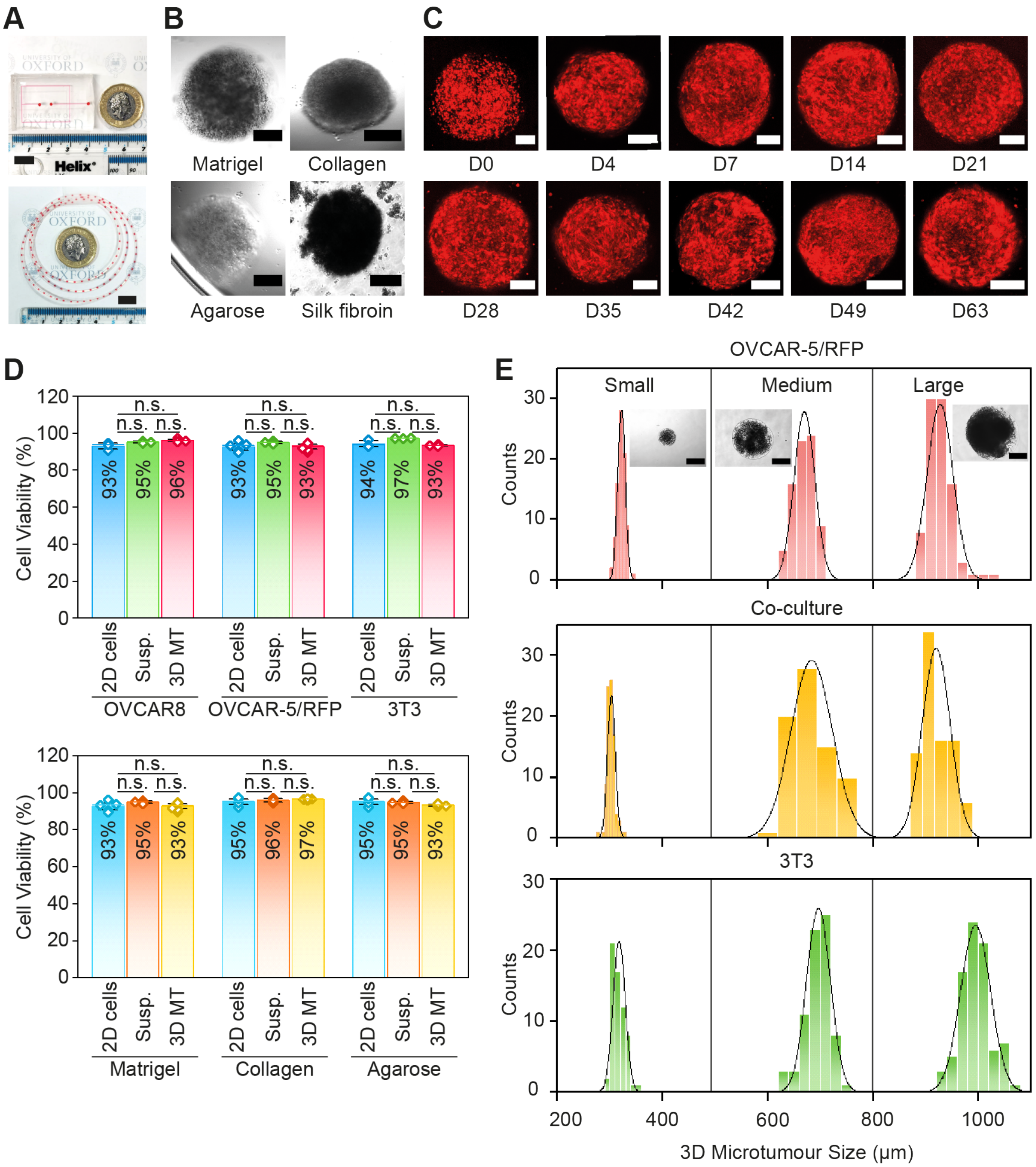

Figure S1. (A) Top: Image of a 3-channel PDMS microfluidic chip. Bottom: Image of 3D microtumors (stained with red dye) in a PTFE exit tube. Scale bar = 1 cm. (B) 3D Microtumors composed of OVCAR8 and different hydrogels. Bright field microscope images for Matrigel, collagen, agarose and silk fibroin microtumors were taken on D0 after fabrication. Scale bar = 300 μm. (C) Long-term culture of 3D microtumors. The 3D microtumors were composed of OVCAR-5/RFP (red) and Matrigel. Confocal microscope images were taken from D0 to D63 after fabrication. Scale bar = 300 μm. (D) Cell viability at three stages of microfluidic fabrication: 2D cells harvested from flasks, cell-hydrogel suspension (Susp.), and 3D microtumors after microfluidic fabrication (3D MT). Top: Matrigel with three cell lines,

OVCAR8, OVCAR-5/RFP and 3T3 fibroblasts. Bottom: OVCAR8 cells with three hydrogels, Matrigel, collagen and agarose. n = 3 to 6. n.s.: not significant, p>0.05. (E) Size distributions of Matrigel 3D microtumors composed of OVCAR-5/RFP tumour cells, co-culture and 3T3 fibroblast cells (n = 62 to 90). Inset: microscope images of 3D microtumors generated with PTFE exit tubes of ID = 300, 650 and 900 µm. Scale bar = 300 µm.

Table S1. Summary of cells used in 3D microtumor fabrication

Cell line	Classification	Source	Catalogue No.
OVCAR-5/RFP	Ovarian cancer	Human	Cell Biolabs Inc. AKR-254
OVCAR5	Ovarian cancer	Human	ATCC
OVCAR8	Ovarian cancer	Human	ATCC
Kuramochi	Ovarian cancer	Human	JCRB CVCL_1345
MDA-MB-231	Breast cancer	Human	Cell Biolabs Inc. AKR-251
HeLa/GFP	Cervical carcinoma	Human	Cell Biolabs Inc. AKR-213
NIH3T3/GFP	Fibroblast	Mouse	Cell Biolabs Inc. AKR-214
3T3-L1 derived adipocytes	Fat cell	Mouse	ATCC CL-173
HEK293T	Embryonic kidney	Human	ATCC CRL-3216

Table S2. Summary of biocompatible hydrogels and their preparation conditions used in 3D microtumor fabrication.

Hydrogels	Preparation for cell-hydrogel suspension	Microfluidic fabrication temperature	Post-fabrication process temperature	Gelation time
Matrigel	Thaw completely on ice, then mix with cells on ice.	8°C	37°C	2 h
Collagen	Thaw completely on ice, then mix with cells on ice.	8°C	37°C	1.5 h
Agarose	Melt at 70°C and cool to 37°C, then mix with cells at 37°C.	37°C	4°C	10 min
Silk fibroin	Thaw completely at 4°C, then mix with cells on ice.	8°C	37°C	30 min

Table S3. Cell viability data at three stages during the microfluidic fabrication of 3D microtumors from various cells and hydrogels (n = 3 to 6).

Cell	Hydrogel	Cell viability		
		2D cells*	Cell-hydrogel suspensions	3D microtumors
OVCAR8	Matrigel	93% ± 2.0%	95% ± 0.58%	96% ± 0.74%
OVCAR-5/RFP	Matrigel	93% ± 2.7%	95% ± 0.85%	93% ± 2.1%
3T3	Matrigel	94% ± 1.9%	97% ± 0.15%	93% ± 0.45%
OVCAR8	Agarose	95% ± 2.0%	95% ± 0.59%	93% ± 0.68%
OVCAR8	Collagen	95% ± 2.0%	96% ± 1.1%	97% ± 0.34%

* 2D cells were harvested from culture flasks.

Table S4. Initial size and time taken for the formation of hypoxic cores in 3D spheroids prepared with different methods.

Fabrication method	Cell type	Initial size (µm)	Time taken for the formation of hypoxic cores	Refere nce
Liquid overlay method	Ewing sarcoma A673	~200	~6 days	[1]
	Lewis lung carcinoma (LLC)	~200	~6 days	
Droplet-Microarray	MCF7	~140	Not characterized	[2]
	HEK293	~160	Not characterized	
	HeLa	~70	Not characterized	
Hanging drop	HT29	~100	21 days	[3]
	HCT116	~100	21 days	
Spinner flask suspension culture	human umbilical cord tissue-derived mesenchymal stromal cells	143 ± 9.78	Not characterized	[4]
3D microtumor by microfluidics	Ovarian cancer OVCAR-5/RFP	670 ± 20<br>930 ± 25	1 day	This work

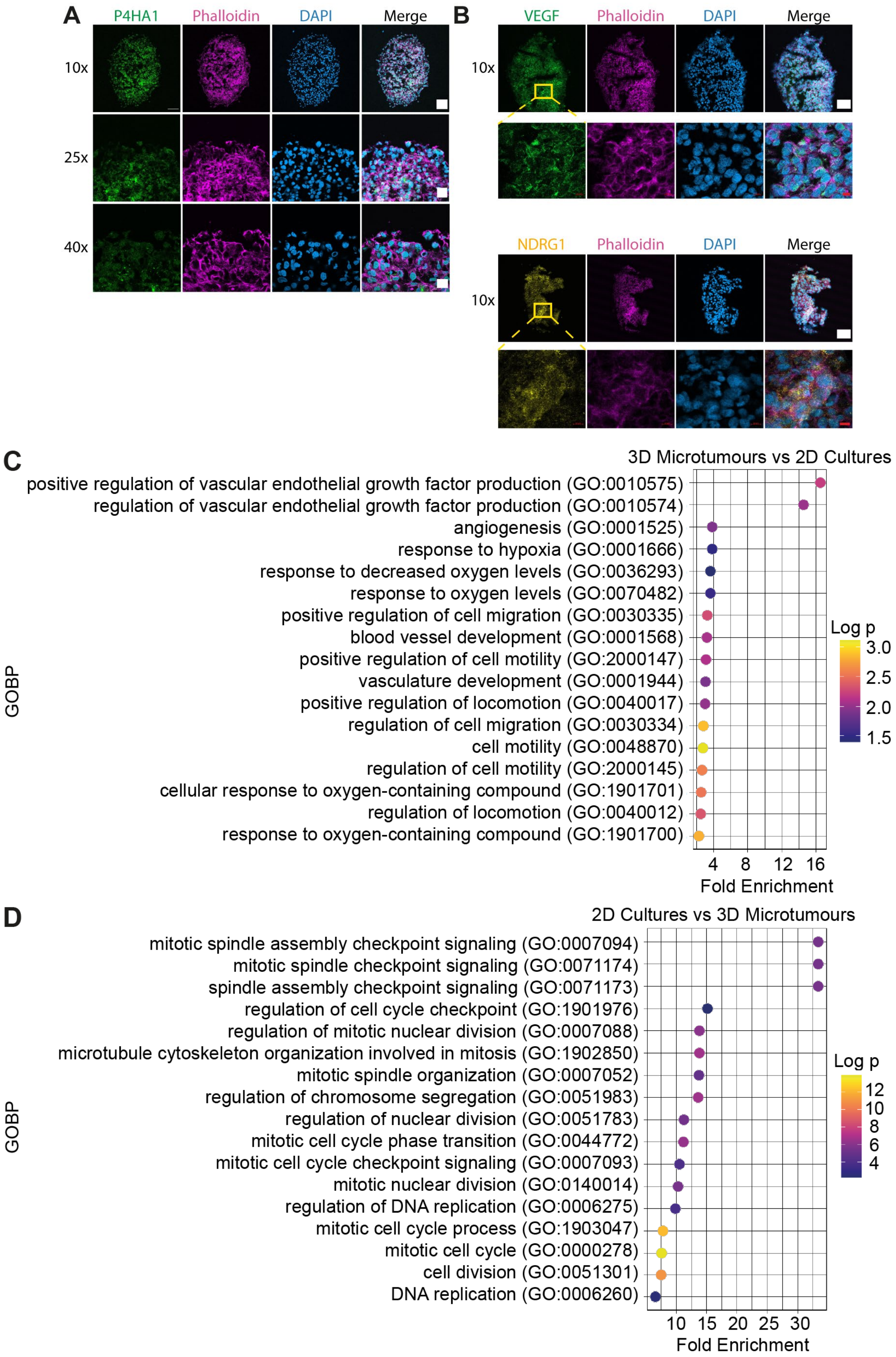
A
P4HA1
Phalloidin
DAPI
Merge
10x
25x
40x
B
VEGF
Phalloidin
DAPI
Merge
10x
NDRG1
Phalloidin
DAPI
Merge
10x
C
3D Microtumours vs 2D Cultures
positive regulation of vascular endothelial growth factor production (GO:0010575)
regulation of vascular endothelial growth factor production (GO:0010574)
angiogenesis (GO:0001525)
response to hypoxia (GO:0001666)
response to decreased oxygen levels (GO:0036293)
response to oxygen levels (GO:0070482)
positive regulation of cell migration (GO:0030335)
blood vessel development (GO:0001568)
positive regulation of cell motility (GO:2000147)
vasculature development (GO:0001944)
positive regulation of locomotion (GO:0040017)
regulation of cell migration (GO:0030334)
cell motility (GO:0048870)
regulation of cell motility (GO:2000145)
cellular response to oxygen-containing compound (GO:1901701)
regulation of locomotion (GO:0040012)
response to oxygen-containing compound (GO:1901700)
GOBP
4 8 12 16
Fold Enrichment
Log p
3.0
2.5
2.0
1.5
D
2D Cultures vs 3D Microtumours
mitotic spindle assembly checkpoint signaling (GO:0007094)
mitotic spindle checkpoint signaling (GO:0071174)
spindle assembly checkpoint signaling (GO:0071173)
regulation of cell cycle checkpoint (GO:1901976)
regulation of mitotic nuclear division (GO:0007088)
microtubule cytoskeleton organization involved in mitosis (GO:1902850)
mitotic spindle organization (GO:0007052)
regulation of chromosome segregation (GO:0051983)
regulation of nuclear division (GO:0051783)
mitotic cell cycle phase transition (GO:0044772)
mitotic cell cycle checkpoint signaling (GO:0007093)
mitotic nuclear division (GO:0140014)
regulation of DNA replication (GO:0006275)
mitotic cell cycle process (GO:1903047)
mitotic cell cycle (GO:0000278)
cell division (GO:0051301)
DNA replication (GO:0006260)
GOBP
10 15 20 25 30
Fold Enrichment
Log p
12
10
8
6
4

Figure S2. (A) Confocal images of 3D microtumors composed of OVCAR5 ovarian cancer cells and Matrigel at D10 stained with P4HA1 (green), phalloidin (pink), DAPI (blue). Scale bar = 100 µm for 10x, 30 µm for 25x, and 20 µm for 40x magnification. (B) Confocal images of 3D microtumors composed of OVCAR5 ovarian cancer cells and Matrigel at D10 stained with VEGFA (green), NDRG1 (yellow), phalloidin (pink), DAPI (blue). Scale bar = 100 µm. Inset: zoom-in images for regions of interest (red scale bar = 10 µm). Dot plots showing the main gene ontology biological processes (GOBP) enriched in (C) 3D microtumors and (D) 2D cultures produced from OVCAR5, OVCAR8 and OVCAR-5/RFP cells.

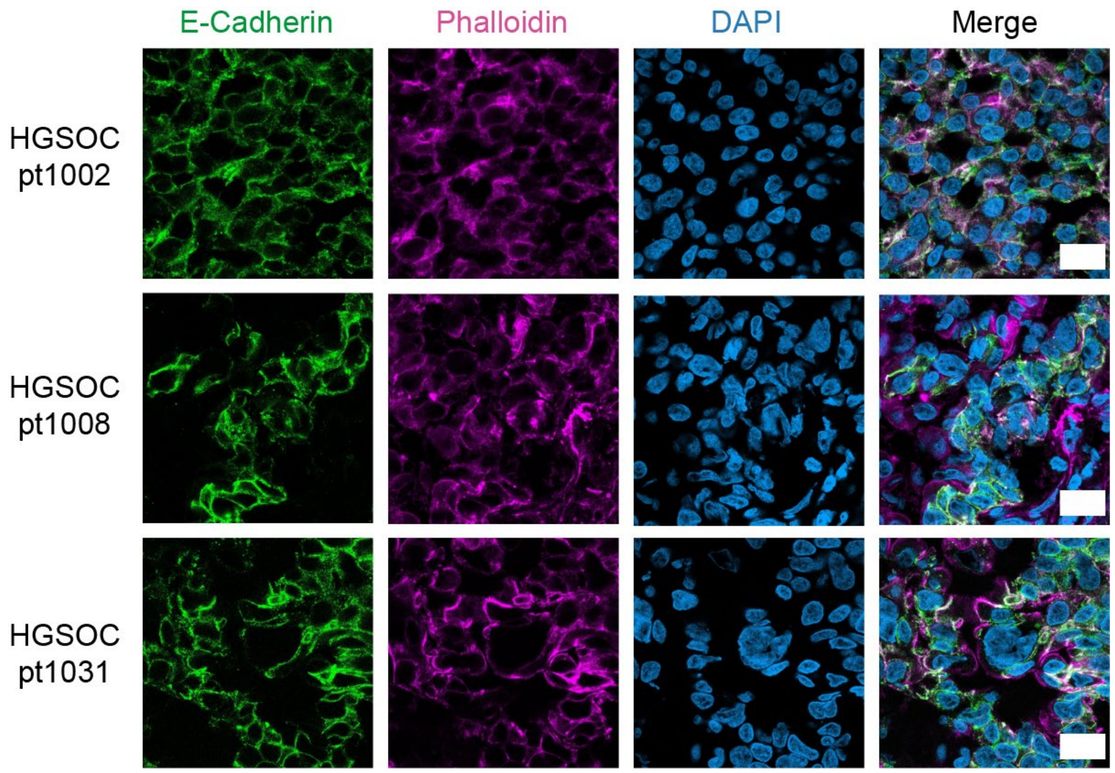

Figure S3. Confocal images of clinical HGSOC samples stained with E-Cadherin (green), phalloidin (pink), DAPI (blue). Scale bar = 20 µm.

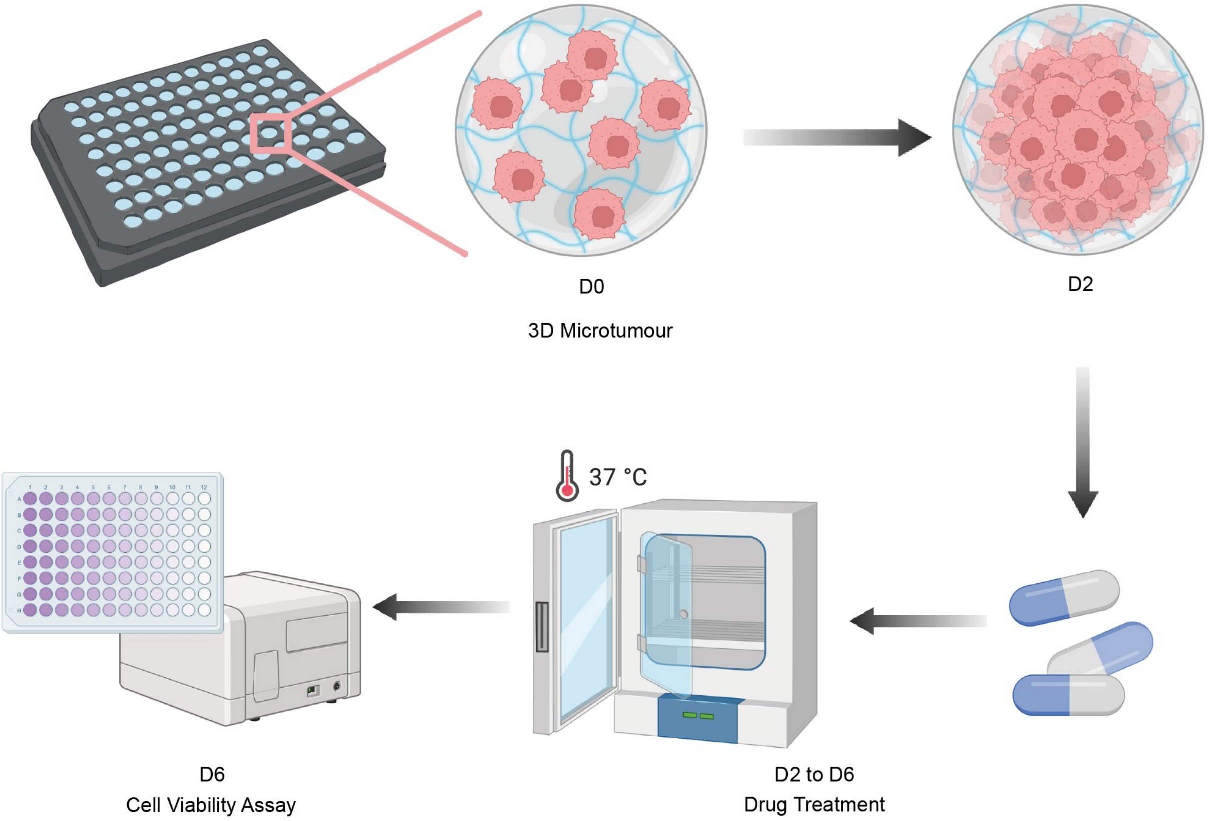

Figure S4. Schematic showing the evaluation of chemotherapeutic responses with 3D microtumors. Each 3D microtumor was seeded to one well of a 96-well plate on D0 of fabrication and kept at 37°C for 2 days. On D2, serial dilutions of chemotherapeutics were applied and the microtumors were cultured at 37°C for 4 days. On D6, a cell viability assay was performed for each 3D microtumor. The diagrams were created with BioRender.com.

Table S5. IC_{50} values calculated from carboplatin and paclitaxel dose-response curves.

	IC_{50}	2D	3D
OVCAR-5/RFP	Carboplatin (μM)	60 ± 6.1	100 ± 12
	Paclitaxel (nM)	2.7 ± 0.9	5.3 ± 2.0
Co-culture	Carboplatin (μM)	37 ± 3.1	110 ± 14
	Paclitaxel (nM)	6.3 ± 0.7	58 ± 27
3T3	Carboplatin (μM)	61 ± 7.6	110 ± 20
	Paclitaxel (nM)	44 ± 18	690 ± 210

Table S6. Calculations for theoretical carboplatin C_{max} and *in vitro* equivalent dose

Theoretical carboplatin plasma C_{max}:	Equivalent *in vitro* dose for carboplatin AUC:
First, consider the average epithelial ovarian cancer patient parameters of age 63, BMI 26, creatinine 0.85 mg dL^{-1} and female gender, and use the Cockcroft-Gault formula to estimate GFR.<br>Next, use the Calvert formula to calculate carboplatin dose: Dose (mg) = AUC (mg mL^{-1})·min x [GFR (mL min^{-1}) + 25 (mL min^{-1})]<br>Assume no excretion and serum-only distribution during the 30-minute infusion window, with a typical blood volume of 4.7 L, to yield the theoretical C_{max} of 280 µM.	*In vitro*, we assume no distribution phase, and a prolonged half-life of 24 h.<br>Using $A = A_0e^{-kt}$ with $A/A_0 = ½$ and t = 1440, we obtain k = ln(2)/1440.<br>Integrating $A = A_0e^{-kt}$ between ∞ and 0, and equating the result to an AUC of 5 (mg mL^{-1})·min, we find A_0 = 5k= 0.0024 mg mL^{-1} = 6.5 µM.

Table S7. Comparison of paclitaxel IC_{50} values of co-culture over OVCAR-5/RFP.

Paclitaxel IC_{50} (nM)	Co-culture	OVCAR-5/RFP	Fold change for IC_{50} value of Co-culture over OVCAR-5/RFP
3D	58 ± 27	5.3 ± 2.0	11
2D	6.3 ± 0.7	2.7 ± 0.9	2.3

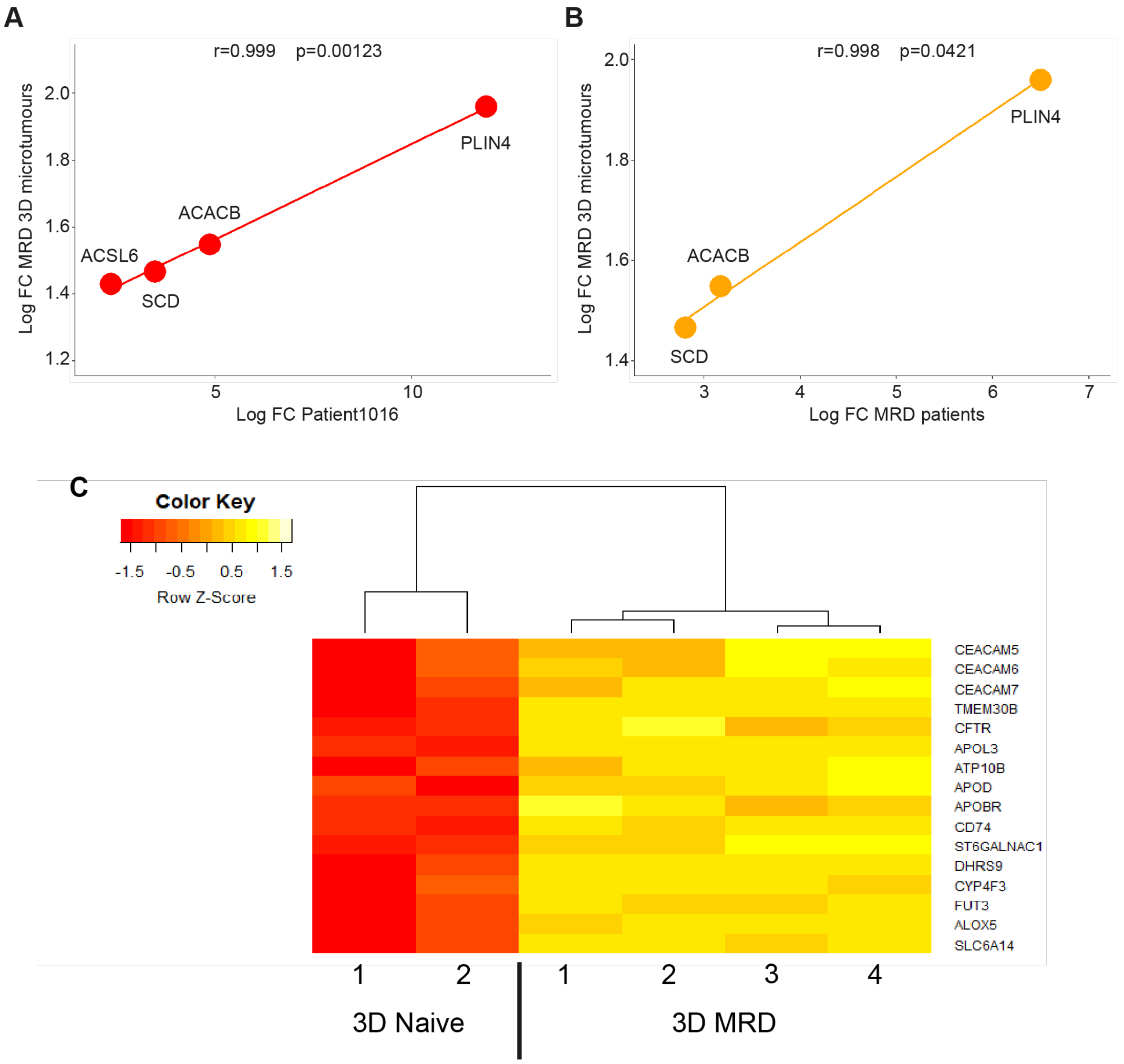

Figure S5. (A, B) Scatterplots showing the $\log_2$ fold change (log FC) of genes involved in lipid metabolism. A positive correlation is observed between their upregulation in the MRD 3D microtumors (3D/2D expression ratios) compared to the MRD patient 1016 (A) or the pooled MRD exceptional responders (B) (post/pre chemo expression ratios). (C) Heatmap showing the expression of lipid-related genes in naïve 3D microtumors and MRD 3D microtumors.

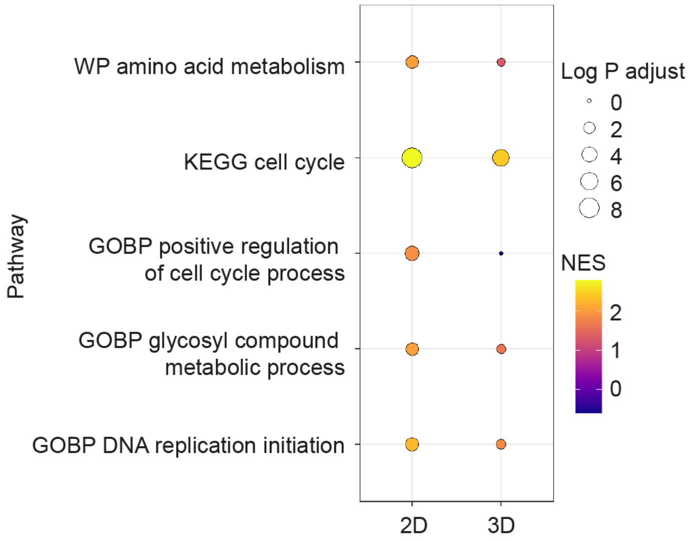

Figure S6. Bubble plots show selected pathways that are upregulated (+ve NES score) in 2D or 3D microtumors relative to clinical samples.

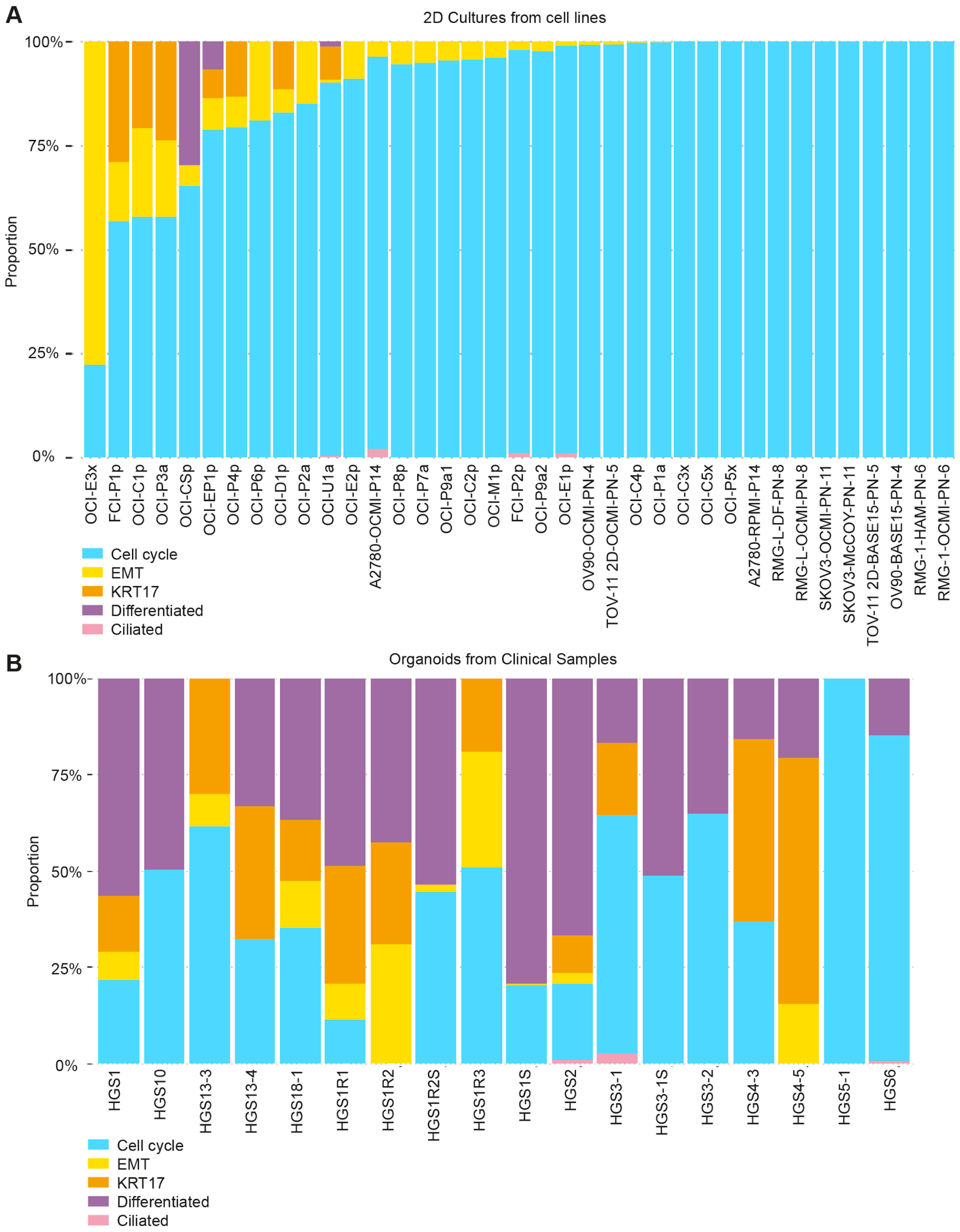

Figure S7. Stacked bar plots visualizing the deconvolution results of (A) a publicly available dataset of 37 additional ovarian cancer cell lines grown in 2D cultures; (B) a publicly available dataset of organoids established from clinical samples. The y axis represents the percentage of each cell state in a given sample. Colours of the bars denote the 5 cell states as shown in the legend.

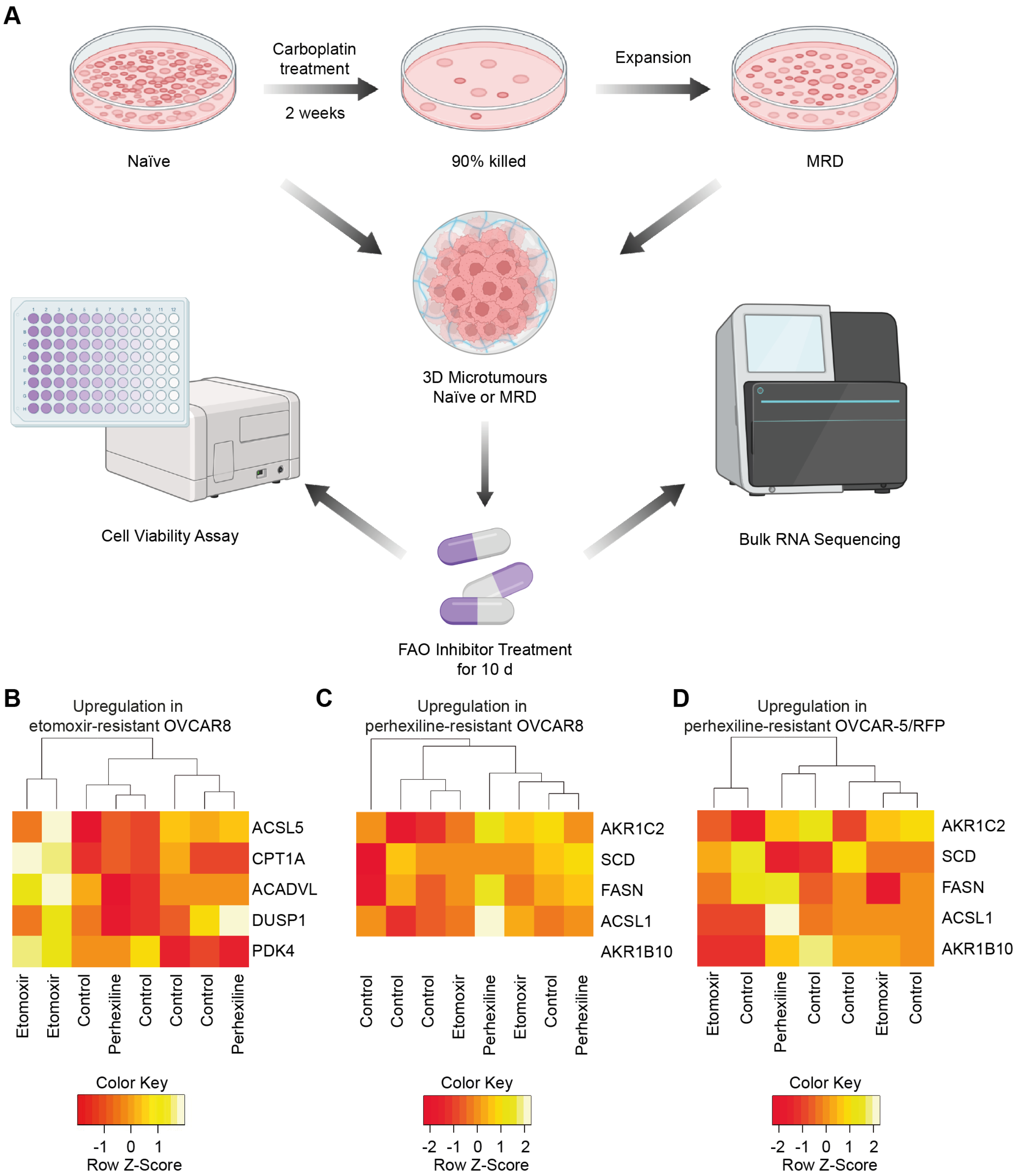

Figure S8. (A) Schematic diagram of the treatment of naïve and MRD cells with chemotherapy agents. The naïve ovarian cancer cells were treated with carboplatin for 2 weeks to achieve 90% cell killing and the surviving MRD cells were collected and expanded. 3D microtumors were fabricated (D0) from naïve cells or the corresponding MRD cells for each cell line. Next, FAO inhibitor treatment was applied to both types of microtumors for 10 days (D0 to D10). Cell viability assays were performed and mRNAs were extracted for bulk RNA sequencing on D10. The diagrams were created with BioRender.com. (B) Heatmap showing differentially expressed genes (DEGs) involved in FAO for etomoxir-resistant MRD 3D microtumors composed of

OVCAR8. Heatmap showing DEGs for perhexiline-resistant MRD 3D microtumors composed of (C) OVCAR8 and (D) OVCAR-5/RFP at D10 (OVCAR8 cells do not express AKR1B10).

Table S8. Killing effect comparison between naïve and MRD 3D microtumors.

Difference in cell viability (compared to DMSO)		Naïve	MRD	Δ = Naïve−MRD
Etomoxir	OVCAR-5/RFP	-1%	-6%	5%
	OVCAR5	10%	12%	-2%
	OVCAR8	30%	-7%	37%
Perhexiline	OVCAR-5/RFP	-15%	-97%	82%
	OVCAR5	-6%	-79%	73%
	OVCAR8	-6%	-54%	48%

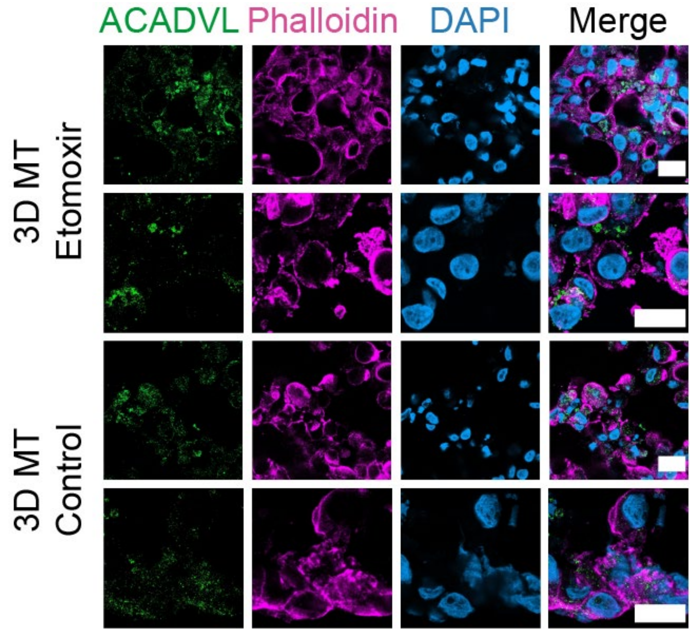

Figure S9. Confocal images of MRD 3D microtumors of FAO markers in 3D microtumors at D10: ACADVL (green). The cells were also stained with phalloidin (pink), DAPI (blue). Scale bar = 20 μm.

Table S9. Protocol for differentiation of 3T3-L1 cells into adipocytes.

Day	Medium
D0	DMEM supplemented with 10% (v/v) bovine calf serum (ATCC, #30-2020) and 1% (v/v) Pen-Strep
D2	DMEM supplemented with 10% (v/v) FBS, 517 µM IBMX (Sigma-Aldrich, #I7018), 1 µM dexamethasone (Sigma-Aldrich, #D2915), and 167 nM insulin (Merck, #407709)
D4	DMEM supplemented with 10% (v/v) FBS and 167 nM insulin
D6	DMEM supplemented with 10% (v/v) FBS

* Cells were maintained at complete confluence for 2 d (D0 to D2).

Table S10. Microplate reader settings for fluorescence intensity measurement to evaluate cell viability.

Parameter	Value
Excitation wavelength	555 nm
Excitation bandwidth	30 nm
Emission wavelength	595 nm
Emission bandwidth	30 nm
Orbital averaging* diameter	4 mm

* With the orbital averaging on, measurements were taken within a circle of 4 mm to give the average fluorescence signal of the area. This setting is favoured in case the 3D microtumor does not sit in the centre of each well.

Table S11. List of antibodies used for immunofluorescence staining.

Antibody	Catalogue number	Dilution
VEGFA	Thermo Fisher MA1-16629	1 in 200
NDRG1	abcam ab124689	1 in 200
P4HA1	abcam ab244302	1 in 200
ALDH3A1	abcam ab76976	1 in 100
SCD	Thermo Fisher MA5-27542	1 in 100
AKR1B10	abcam ab96417	1 in 500
CPT1A	abcam ab128568	1 in 50
ACADVL	abcam ab155138	1 in 200
AKR1C1/2	abcam ab131375	1 in 200
FASN	abcam ab128856	1 in 50

References

[1] S. Riffle, R. N. Pandey, M. Albert, R. S. Hegde, *BMC Cancer* **2017**, 17, 338.

[2] A. A. Popova, T. Tronser, K. Demir, P. Haitz, K. Kuodyte, V. Starkuviene, P. Wajda, P. A. Levkin, *Small* **2019**, 15, 1901299.

[3] S. Däster, N. Amatruda, D. Calabrese, R. Ivanek, E. Turrini, R. A. Droeser, P. Zajac, C. Fimognari, G. C. Spagnoli, G. Iezzi, V. Mele, M. G. Muraro, *Oncotarget* **2016**, 8, 1725.

[4] J. M. Santos, S. P. Camões, E. Filipe, M. Cipriano, R. N. Barcia, M. Filipe, M. Teixeira, S. Simões, M. Gaspar, D. Mosqueira, D. S. Nascimento, P. Pinto-do-Ó, P. Cruz, H. Cruz, M. Castro, J. P. Miranda, *Stem Cell. Res. Ther.* **2015**, 6, 90.
